# Synthesis and Biological Evaluation of Novel 2-Methoxypyridylamino-Substituted Riminophenazine Derivatives as Antituberculosis Agents

**DOI:** 10.3390/molecules19044380

**Published:** 2014-04-09

**Authors:** Dongfeng Zhang, Yang Liu, Chunlin Zhang, Hao Zhang, Bin Wang, Jian Xu, Lei Fu, Dali Yin, Christopher B. Cooper, Zhenkun Ma, Yu Lu, Haihong Huang

**Affiliations:** 1 State Key Laboratory of Bioactive Substance and Function of Natural Medicines & Beijing Key Laboratory of Active Substances Discovery and Druggability Evaluation, Institute of Materia Medica, Peking Union Medical College & Chinese Academy of Medical Sciences, 1 Xian Nong Tan Street, Beijing 100050, China; E-Mails: zdf@imm.ac.cn (D.Z.); liuyang3923@163.com (Y.L.); chunlin.zhang@hotmail.com (C.Z.); hubert365@163.com (H.Z.); yindali@imm.ac.cn (D.Y.); 2 Department of Pharmacology, Beijing Tuberculosis and Thoracic Tumor Research Institute, Beijing Chest Hospital, Capital Medical University, 97 Ma Chang Street, Beijing 101149, China; E-Mails: binbin69823233@126.com (B.W.); xujian4614@gmail.com (J.X.); fl2000x@163.com (L.F.); 3 Global Alliance for TB Drug Development, 40 Wall Street, New York, NY 10005, USA; E-Mail: christopher.cooper@tballiance.org

**Keywords:** clofazimine, antituberculosis activity, 2-methoxypyridylamino-substituted riminophenazines, skin discoloration

## Abstract

Clofazimine, a member of the riminophenazine class, is one of the few antibiotics that are still active against multidrug-resistant *Mycobacterium tuberculosis* (*M. tuberculosis*). However, the clinical utility of this agent is limited by its undesirable physicochemical properties and skin pigmentation potential. With the goal of maintaining potent antituberculosis activity while improving physicochemical properties and lowering skin pigmentation potential, a series of novel riminophenazine derivatives containing a 2-methoxypyridylamino substituent at the C-2 position of the phenazine nucleus were designed and synthesized. These compounds were evaluated for antituberculosis activity against *M. tuberculosis* H37Rv and screened for cytotoxicity. Riminophenazines bearing a 3-halogen- or 3,4-dihalogen-substituted phenyl group at the N-5 position exhibited potent antituberculosis activity, with MICs ranging from 0.25~0.01 μg/mL. The 3,4-dihalogen- substituted compounds displayed low cytotoxicity, with IC_50_ values greater than 64 μg/mL. Among these riminophenazines, compound **15** exhibited equivalent *in vivo* efficacy against *M. tuberculosis* infection and reduced skin discoloration potential in an experimental mouse infection model as compared to clofazimine. Compound **15**, as compared to clofazimine, also demonstrated improved physicochemical properties and pharmacokinetic profiles with a short half-life and less drug tissue accumulation. This compound is being evaluated as a potential drug candidate for the treatment of multidrug resistant tuberculosis.

## 1. Introduction

Tuberculosis (TB) has become a global public health emergency due to the rapid development of multidrug-resistant tuberculosis (MDR-TB). According to the Global Tuberculosis Report released by World Health Organization in 2013, an estimated 8.6 million people developed TB and 1.3 million people died from TB in 2012, including 320,000 deaths among HIV-positive people [1]. Some progress has been made to develop new TB drugs. Currently, the global TB drug pipeline consists of about 10 new or repurposed drugs in Phase II or Phase III clinical trials. Bedaquiline, a new TB drug with a novel mechanism of action has become the first drug approved by the US Food and Drug Administration (FDA) for the treatment MDR-TB in more than 40 years [1,2,3]. Despite the progress, there is still an urgent need for new and more effective therapy for the treatment MDR-TB and repurposing or optimizing existing antibiotics has been proved an effective approach in identifying new TB drugs [2,4].

**Figure 1:**
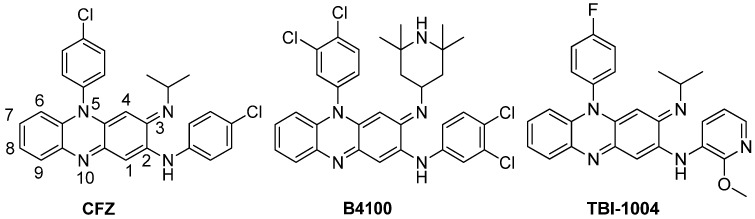
Structures of CFZ and its analogues.

Clofazimine (CFZ, Figure 1) was first introduced in 1960s for the treatment of leprosy [5]. This agent has demonstrated excellent activity against MDR-TB both *in vitro* and *in vivo*, and has been used in clinical trials for the treatment of MDR-TB [6]. We have conducted a systematic structural modification on CFZ to address the skin discoloration problem, which is one of the major obstacles for its clinical use [7,8,9]. We have identified a novel series of riminophenazine derivatives containing a 2-methoxypyridylamino substituent at the C-2 position of the phenazine nucleus. This series of compounds, exemplified by TBI-1004 (Figure 1), displayed potent antituberculosis activity, reduced lipophilicity, and improved pharmacokinetic profiles as compared to CFZ [8]. We also concluded that an electron-withdrawing group at the para-position of the phenyl ring at the N-5 position was beneficial to antituberculosis activity [9]. Previously, O’Sullivan and coworkers found that compoundsbearing a 3,4-dichlorophenyl or 3,4,5-trichlorophenyl group at the N-5 position and a tetramethylpiperidyl group at the C-3 position, such as compound B4100 (Figure 1), possessed improved antituberculosis activity as compared to CFZ [10,11]. These work prompted us to further investigate the effect of the halogen atoms on the phenyl ring at the N-5 position for antituberculosis activity based on our 2-methoxypyridylamino-substituted riminophenazine system.

The present work aimed to identify novel riminophenazine derivatives with potent antituberculosis activity, improved physicochemical property and pharmacokinetic profiles and low skin pigmentation potential. Hence, we kept the phenazine nucleus with the 2-methoxypyridylamino group at the C-2 position intact. Our previous work indicated that the 2-methoxypyridylamino group is a privileged moiety [9]. We focused our investigation on the halogen effect of the N-5 phenyl group. In addition, we introduced cyclic alkyl group including *O*-containing heterocyclic group to the imino nitrogen of CFZ and evaluated their impact on antituberculosis activity and lipophilicity (ClogP).

## 2. Results and Discussion

### 2.1. Chemistry

**Scheme 1:**
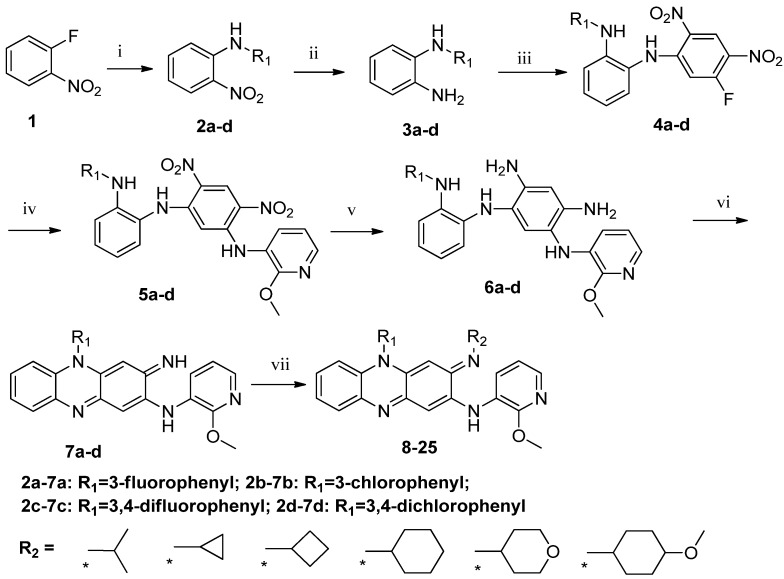
Synthesis of the target compounds **8**–**25**.

The synthetic route for target compounds **8**–**25** is illustrated in Scheme 1. The most compounds were prepared based on the previously published protocols [9,12]. Nitro compounds **2a**–**d** were synthesized from commercially available 2-fluoronitrobenzene (**1**) and aryl amines via aromatic nucleophilic substitution. The nitro group in compounds **2a**–**d** was reduced to give amine compounds **3a**–**d**, and followed by substitution with 1,5-difluoro-2,4-dinitrobenzene (DFDNB) to afford compounds **4a**–**d**. The formation of the key intermediates **5a**–**d** was achieved by aromatic nucleophilic displacement in good yields. Reduction of nitro groups in **5a**–**d** was carried out using different methods depending on the structure of the substrate. For examples, compounds **5a** and **5c** were reduced by using catalytic hydrogenation, while compounds **5b** and **5d** were reduced by using zinc powder and glacial acetic acid. Compounds **6a**–**d** underwent spontaneous cyclization to afford riminophenazines **7a**–**d**. Final target compounds **8**–**25** were prepared by replacing the imines compounds **7a**–**d** with different amine side chains in the presence of glacial acetic acid. 

### 2.2. Biological Results and Discussion

Table 1 summarizes the structure and biological data for 18 new riminophenazines. CFZ and TBI-1004 were included as reference compounds. All new compounds were screened for *in vitro* activity against *M. tuberculosis* H37Rv using the Microplate Alamar Blue Assay (MABA) in 96-well plate format. The antibacterial activity of the compounds is indicated by minimum inhibitory concentration (MIC) values. The target compounds were also tested for cytotoxicity using Vero cells measured as a concentration inhibiting 50% growth (IC_50_) as compared to a no treatment control [8]. The lipophilicity is estimated by ClogP, calculated by ChemOffice 2004 software.

*Reagents and conditions*: (i) KF, R_1_NH_2_, 160 °C, 24%–76%; (ii) 10% Pd/C, H_2_, ethanol or Zn/CH_3_COOH, THF, rt; (iii) DFDNB, Et_3_N, ethanol, rt, 30%–94% (two steps from **2a**–**d**); (iv) 3-amino-2-methoxypyridine, Et_3_N, THF, reflux, 51%–88%; (v) 10% Pd/C, methanol, THF, H_2_ or Zn/CH_3_COOH, CH_2_Cl_2_, rt; (vi) air, methanol, rt, 24%–94% (two steps from **5a**–**d**); (vii) R_2_NH_2_, CH_3_COOH, dioxane, sealed tube, 110 °C, 33%–86%.

Lipophilicity (ClogP) is an important property for riminophenazine derivatives, as skin discoloration side effect of CFZ is closely related to its high lipophilicity [13]. As shown in Table 1, the ClogP values for the most new compounds are lower than that of CFZ (7.50), ranging from 4.70 to 7.17 with the exception of compound **13**. Compounds with a 3-fluoro- or 3,4-difluorophenyl group at the N-5 position displayed significantly reduced lipophilicity as compared to the corresponding chloro substituted compounds, as exemplified by compound **8** (ClogP 5.90) *versus* compound **10** (ClogP 6.47), compound **15** (ClogP 5.98) *versus* compound **21** (ClogP 7.07). These results are consistent with previous observation that chlorination of CFZ and its analogues improves antibacterial activity and increases lipophilicity, which is a major factor for skin pigmentation [14]. In addition, the tetrahydropyranyl substituent on the imino nitrogen at the C-3 position, as compared to the other groups, significantly reduces ClogP value, as exemplified by compound **14** (ClogP 5.27) *versus* compound **10** (ClogP 6.47), compound **19** (ClogP 4.77) *versus* compound **15** (ClogP 5.98). This substituent could serve as an interesting lead for identifying new analogues with further decreased lipophilicity [15].

**Table 1:** Physicochemical property (ClogP), antituberculosis activity (MIC), cytotoxicity (VERO IC_50_), and selectivity index (SI) values for target compounds. 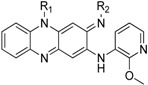

Compound	R_1_	R_2_	ClogP ^a^	MIC(μg/mL)	IC_50_(μg/mL)	SI ^b^
CFZ			7.50	0.12	68.6	572
TBI-1004	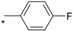	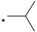	5.90	0.038	>64	1684
**8**	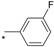	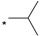	5.90	0.06	2.65	44
**9**	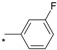	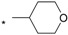	4.70	0.061	9.21	151
**10**	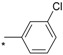	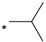	6.47	0.250	5.10	20
**11**	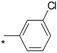	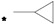	5.99	0.030	50.88	1696
**12**	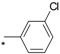	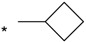	6.55	0.060	>64	1067
**13**	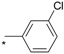	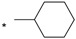	7.67	0.057	53.26	934
**14**	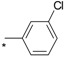	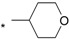	5.27	0.025	23.40	936
**15**	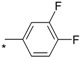	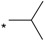	5.98	0.066	>64	970
**16**	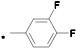	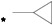	5.50	0.014	25.51	1822
**17**	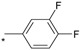	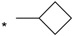	6.06	0.040	>64	1600
**18**	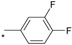	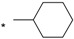	7.17	0.030	>64	2133
**19**	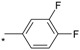	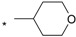	4.77	0.067	>64	955
**20**	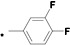	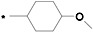	5.80	0.056	>64	1143
**21**	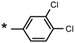	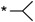	7.07	0.099	>64	646
**22**	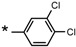	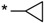	6.59	0.025	>64	2560
**23**	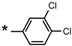	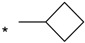	7.15	0.06	>64	1067
**24**	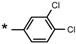	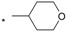	5.86	0.080	>64	800
**25**	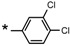	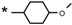	6.89	0.059	>64	1085

^a^ ClogP values, calculated using ChemOffice 2004 software. ^b^ SI = selectivity index IC_50_/MIC (For IC_50_ values of >64 μg/mL, a value of 64 μg/mL was used for the SI calculation).

Against *M. tuberculosis* H37Rv, the majority of compounds exhibited potent activity equal or better than CFZ and TBI-1004 with MIC values ranging from 0.01 to 0.1 μg/mL. It appears that the number, type and position of the halogen atoms on the phenyl ring at the N-5 position have no significant impact on the antituberculosis activity. However, compounds with a 3-F or 3-Cl substituted phenyl group on the N-5 position have showed higher cytotoxicity as compared to its corresponding 4- and 3,4-disubstituted compounds, as exemplified by compound **8** (IC_50_ 2.65 μg/mL) *versus* TBI-1004 (IC_50_ > 64 μg/mL). Interestingly, all compounds with a 3,4-dihalogen-substituted phenyl groups displayed very low cytotoxicity, with IC_50_ > 64 μg/mL, with the exception of compound **16** (IC_50_ 25.51 μg/mL).

Based on our systematic evaluation of lipophilicity, antituberculosis activity and cytotoxicity, two compounds **15** and **22** were selected for a multiple doses *in vivo* toxicity study in mice. Table 2 summarizes the tolerability data by recording the number of mice which survived after an oral administration at a dose of 500 mg/kg, once daily for 4 days, followed by a 7-day observation. As shown in Table 2, both compounds **15** and **22** demonstrated good safety in mice. Therefore, the *in vivo* efficacy evaluation of compounds **15** and **22** were conducted in a mouse model infected by *M. tuberculosis* H37Rv. The *in vivo* efficacy results were summarized in Table 3. Compound **15** demonstrated excellent efficacy in mice, the bacterial burden in the lungs was reduced by 3.8 logs colony forming units (CFU) as compared to the untreated control group and equivalent to CFZ. Furthermore, during the *in vivo* study, we also observed that the discoloration of the ears, internal organs and fat tissues of the mice treated with compound **15** appeared less intense as compared to CFZ. However, compound **22** failed to display efficacy in mouse model. The inconsistency between the *in vivo* and *in vitro* results might be explained by poor pharmacokinetics [16]. Finally, preliminary PK in mice was evaluated for compound **15**. As shown in Table 4, compound **15** demonstrated a shorter plasma half-life (t_1/2_), higher Cmax and AUC as compared to CFZ, which could explain its excellent efficacy and reduced skin discoloration potential compared to CFZ.

**Table 2:** Preliminary multidose toxicity study of compounds **15** and **22 **^a^.

Compound	Number of animals that survived/Total number of animals
**15**	5/5
**22**	6/6

^a^ oral administration at a dose of 500 mg/kg, once daily for 4 days, followed by 7-day observation.

**Table 3:** Efficacy of CFZ, compounds **15** and **22** after 30 days of treatment of BALB/c mice infected with *M. tuberculosis* H37Rv. (mean ± SD) ^a^.

Groups	Dose (mg/kg)	logCFU/lung
Control (D3)		1.94 ± 028
Control (D10)		3.72 ± 0.46
Control (D30)		8.32 ± 0.19
CFZ	20	4.29 ± 0.58
**15**	20	4.56 ± 0.38
**22**	20	8.06 ± 0.13

^a^ 5 mice were used in every group (n = 5).

**Table 4:** Preliminary pharmacokinetic parameters of CFZ and **15** dosed orally in mice at 20 mg/kg.

Compound	T_1/2_ (h)	Tmax (h)	Cmax (mg/L)	AUC_0~24 h_ (mg/L*h)
CFZ	27.11	2	0.47	8.22
**15**	13.80	2	1.835	21.156

## 3. Experimental

### 3.1. General Information

#### 3.1.1. Chemistry

All reagents and solvents were purchased from commercial sources unless otherwise indicated. Melting points were determined on Yanaco MP-J3 melting point apparatus. Thin-layer chromatography was performed with fluorescent silica gel plates GF254, which were checked under UV (254 nm) light. ^1^H-NMR spectra were recorded on Varian mercury-300 MHz or Varian-400 MHz NMR spectrometer in CDCl_3_ or DMSO-*d_6_*. ^13^C-NMR spectra were obtained on Varian-400 at 100 MHz in CDCl_3_. High-resolution mass spectra were measured on an Agilent 1100 series LC/MSD trap mass spectrometer (ESI-TOF).

#### 3.1.2. Minimum Inhibitory Concentration and Cytotoxicity Assays

These were carried out according to our published protocols [8].

#### 3.1.3. *In vivo* Acute *M. tuberculosis* H37Rv Infection Assay and Mouse Pharmacokinetic Study

These were carried out according to our published protocols [8].

### 3.2. General Procedure for Preparation of Compounds **2a**–**d**

*N-(3-Fluorophenyl)-2-nitroaniline* (**2a**). A mixture of 2-fluoronitrobenzene (21.2 g, 150 mmol), 3-fluoroaniline (20.0 g, 180 mmol) and anhydrous potassium fluoride (8.7 g, 150 mmol) was stirred at 160 °C for 36 h. The mixture was then cooled, water and ethyl acetate were added, the aqueous layer was extracted with ethyl acetate, and the combined organic layer was washed with 2 N HCl and dried over anhydrous sodium sulfate. After filtration, the filtrate was concentrated under vacuum, and the crude product was recrystallized in 60 mL of 95% ethanol to give 17.4 g of **2a** as a red solid in 50% yield. Mp: 73–74 °C. ^1^H-NMR (300 MHz, CDCl_3_) δ: 6.82–6.94 (2 H, m), 6.99–7.07 (2 H, m), 7.29–7.45 (3 H, m), 8.21 (1 H, d, *J* = 8.4 Hz), 9.43 (1 H, s).

*N-(3-Chlorophenyl)-2-nitroaniline* (**2b**). Compound **2b** was synthesized in a similar manner as described for **2a** to provide 18.7 g of a red solid, yield 76%. Mp: 88–90 °C. ^1^H-NMR (300 MHz, DMSO-*d_6_*) δ: 6.98 (1 H, m), 7.19 (1 H, m), 7.28 (2 H, m), 7.37 (2 H, m), 7.56 (1 H, m), 8.12 (1 H, dd, *J* = 8.1, 1.5 Hz), 9.28 (1 H, brs).

*N-(3,4-Difluorophenyl)-2-nitroaniline* (**2c**). Compound **2c** was synthesized in a similar manner as described for **2a** to give 23.1 g of a red solid, yield 61%. Mp: 95–97 °C.^1^H-NMR (300 MHz, CDCl_3_) δ: 6.84 (1 H, t, *J* = 7.5 Hz), 6.99–7.03 (1 H, m), 7.10–7.17 (2 H, m), 7.22 (1 H, d, *J* = 9.0 Hz), 7.41 (1 H, m), 8.22 (1 H, dd, *J* = 8.4, 1.2 Hz), 9.35 (1 H, s).

*N-(3,4-Dichlorophenyl)-2-nitroaniline* (**2d**). Compound **2d** was synthesized in a similar manner as described for **2a** to afford 20.0 g of a red solid, yield 24%. Mp: 78–79 °C. ^1^H-NMR (300 MHz, CDCl_3_) δ: 6.87 (1 H, d, *J* = 6.9 Hz), 7.12 (1 H, d, *J* = 8.4 Hz), 7.22 (1 H, s), 7.41 (1 H, d, *J* = 6.9 Hz), 7.44–7.48 (2 H, m), 8.21 (1 H, d, *J* = 8.4 Hz), 9.47 (1 H, s).

### 3.3. General Procedure for Preparation of Compounds **4a**–**d**

*1-[2-(3-Fluoroanilino)anilino]-3-fluoro-4,6-dinitrobenzene* (**4a**). Zinc powder (40 g, 615 mmol) was added portionwise into a mixture of **2a** (17 g, 73 mmol) in THF (250 mL) and glacial acetic acid (18 mL, 315 mmol) at room temperature. After filtration, DFDNB (14.9 g, 73 mmol) and triethylamine (7.4 g, 73 mmol) were added to the filtrate (**3a**); the mixture was stirred at room temperature for 9 h, and was concentrated to dryness. The residue was washed with ethanol and filtered to give 19.81 g of **4a** in 70% yield as a red solid. Mp: 170–171 °C. ^1^H-NMR (300 MHz, DMSO-*d_6_*) δ: 6.50 (1 H, d, *J* = 14.1 Hz), 6.60 (1 H, t, *J* = 8.7 Hz), 6.69–6.78 (2 H, m), 7.08–7.22 (2 H, m), 7.32–7.38 (3 H, m), 7.99 (1 H, s), 8.88 (1 H, d, *J* = 7.5 Hz), 9.99 (1 H, s).

*1-[2-(3-Chloroanilino)anilino]-3-fluoro-4,6-dinitrobenzene* (**4b**). Compound **4b** was synthesized in a similar manner as described for **4a** to provide 1.21 g of an orange solid, yield 30%. Mp: 145–147 °C. ^1^H-NMR (300 MHz, DMSO-*d_6_*) δ: 6.49 (1 H, d, *J* = 14.1 Hz), 6.82 (1 H, d, *J* = 7.5 Hz), 6.90 (2 H, m), 7.12 (2 H, m), 7.34 (3 H, m), 7.97 (1 H, s), 8.87 (1 H, d, *J* = 8.1 Hz), 9.97 (1 H, s).

*1-[2-(3,4-Difluoroanilino)anilino]-3-fluoro-4,6-dinitrobenzene* (**4c**). A mixture of **2c** (23 g, 91.9 mmol) and 10% Pd/C (2.3 g) in ethanol was shaken at room temperature under a hydrogen atmosphere (40 psi) for 2 h. After filtration, DFDNB (18.75 g, 91.9 mmol) and triethylamine (9.29 g, 91.9 mmol) were added to the filtrate (**3c**); the mixture was stirred at room temperature for 15 h, filtered, and washed with ethanol to give 34.82 g of **4c** in 94% yield as an orange solid. Mp: 185–186 °C. ^1^H-NMR (300 MHz, CDCl_3_) δ: 5.59 (1 H, s), 6.65 (1 H, d, *J* = 12.9 Hz), 6.72 (1 H, d, *J* = 9.0 Hz), 6.83–6.90 (1 H, m), 7.04–7.13 (2 H, m), 7.29–7.37 (3 H, m), 9.17 (1 H, d, *J* = 7.5 Hz), 9.60 (1 H, s).

*1-[2-(3,4-Dichloroanilino)anilino]-3-fluoro-4,6-dinitrobenzene* (**4d**). Compound **4d** was synthesized in a similar manner as described for **4a** to give 5.9 g of an orange solid, yield 65%. Mp: 210–211 °C. ^1^H-NMR (300 MHz, CDCl_3_) δ: 5.60 (1 H, s), 6.47 (1 H, d, *J* = 13.2 Hz), 6.83 (1 H, dd, *J* = 8.7, 2.4 Hz), 7.07 (1 H, d, *J* = 2.7 Hz), 7.12–7.17 (1 H, m), 7.29–7.37 (4 H, m), 9.14 (1 H, d, *J* = 8.1 Hz), 9.61 (1 H, s).

### 3.4. General Procedure for Preparation of Compounds **5a**–**d**

*1-[2-(3-Fluoroanilino)anilino]-3-(2-methoxy-3-pyridyl)amino-4,6-dinitrobenzene* (**5a**). A mixture of **4a** (3.09 g, 8 mmol), 3-amino-2-methoxypyridine (0.99 g, 8 mmol), triethylamine (0.81 g, 8 mmol) and THF (50 mL) was refluxed for 31 h. After being cooled to room temperature, the mixture was concentrated *in vacuo*, CH_3_OH was added to the residue, and the solid obtained was filtered to give 3 g of **5a** as a red solid in 77% yield. Mp: 180–182 °C. ^1^H-NMR (400 MHz, CDCl_3_) δ: 3.92 (3 H, s), 5.78 (1 H, s), 6.19 (1 H, s), 6.62 (3 H, m,), 6.70–6.85 (1 H, m), 6.99 (1 H, t, *J* = 7.4 Hz), 7.09–7.27 (3 H, m), 7.47–7.29 (2 H, m), 7.98 (1 H, d, *J* = 4.6 Hz), 9.30 (1 H, s), 9.40 (1 H, s), 9.68 (1 H, s).

*1-[2-(3-Chloroanilino)anilino]-3-(2-methoxy-3-pyridyl)amino-4,6-dinitrobenzene* (**5b**). Compound **5b** was synthesized in a similar manner as described for **5a** to give 2.59 g of a red solid, yield 51%. Mp: 151–153 °C. ^1^H-NMR (300 MHz, CDCl_3_) δ: 3.80 (3 H, s), 5.82 (1 H, s), 6.68 (1 H, dd, *J* = 8.1, 1.5 Hz), 6.77 (1 H, brs), 6.81 (1 H, dd, *J* = 8.1, 1.5 Hz), 6.98 (2 H, m), 7.14 (1 H, t, *J* = 8.1 Hz), 7.22 (3 H, m), 7.53 (1 H, dd, *J* = 7.8, 1.5 Hz), 7.88 (1 H, brs), 8.03 (1 H, dd, *J* = 8.1, 1.5 Hz), 9.01 (1 H, s), 9.51 (1 H, brs),9.56 (1 H, brs).

*1-[2-(3,4-Difluoroanilino)anilino]-3-(2-methoxy-3-pyridyl)amino-4,6-dinitrobenzene* (**5c**). Compound **5c** was synthesized in a similar manner as described for **5a** to afford 8.92 g of an orange solid, yield 88%. Mp: 201–203 °C. ^1^H-NMR (300 MHz, DMSO-*d_6_*) δ: 3.78 (3 H, s), 5.76 (1 H, s), 6.53 (1 H, d, *J* = 8.7 Hz), 6.66–6.73 (1 H, m), 6.92–6.97 (2 H, m), 7.13–7.23 (4 H, m), 7.52 (1 H, d, *J* = 7.5 Hz), 7.78 (1 H, s), 8.01 (1 H, d, *J* = 5.1 Hz), 9.00 (1 H, s), 9.49 (1 H, s), 9.54 (1 H, s).

*1-[2-(3,4-Dichloroanilino)anilino]-3-(2-methoxy-3-pyridyl)amino-4,6-dinitrobenzene* (**5d**). Compound **5d** was synthesized in a similar manner as described for **5a** to give 4.0 g of a yellow solid, yield 74%. Mp: 220–221 °C. ^1^H-NMR (300 MHz, DMSO-*d_6_*) δ: 3.83 (3 H, s), 5.76 (1 H, s), 6.66 (1 H, d, *J* = 7.8 Hz), 6.93 (1 H, d, *J* = 5.1 Hz), 6.99–7.03 (2 H, m), 7.23–7.32 (4 H, m), 7.51 (1 H, d, *J* = 6.6 Hz), 7.95 (1 H, s), 8.01 (1 H, d, *J* = 6.6 Hz), 8.99 (1 H, s), 9.49 (1 H, s), 9.54 (1 H, s).

### 3.5. General Procedure for Preparation of Compounds **7a**–**d**

*5-(3-Fluorophenyl)-3-imino-2-(2-methoxy-3-pyridyl)amino-3,5-dihydrophenazine* (**7a**). A mixture of **5a** (2.99 g, 6.1 mmol) and 10% Pd/C (0.3 g) in methanol and THF was shaken at room temperature under a hydrogen atmosphere (40 psi) for 15 h. After filtration, the filtrate was concentrated under vacuum and the residue **6a** was dissolved in methanol. The result solution stirred at room temperature under air for 18 h. The mixture was filtered to give 1.72 g crude **7a** as a red solid in 69% yield. Compound **7a** was taken to the next step without further purification.

*5-(3-Chlorophenyl)-3-imino-2-(2-methoxy-3-pyridyl)amino-3,5-dihydrophenazine* (**7b**). Zinc powder (1.63 g, 25 mmol) was added portionwise into a mixture of **5b** (2.53 g, 5 mmol) and glacial acetic acid (0.86 mL, 15 mmol) in CH_2_Cl_2_ (300 mL) at room temperature. The mixture was stirred until the color turned to light green and then filtered and washed with CH_2_Cl_2_. The filtrate was concentrated, and the residue was treated with water and made alkaline with ammonia. The solid was filtered, washed with water, and then dissolved in anhydrous methanol. The solution was stirred under air overnight. The solid formed was filtered to give 2.01 g crude **7b** in 94% yield, which was taken to the next step without further purification.

*5-(3,4-Difluorophenyl)-3-imino-2-(2-methoxy-3-pyridyl)amino-3,5-dihydrophenazine* (**7c**). Compound **7c** was synthesized in a similar manner as described for **7a** to give 6.46 g of a red solid, yield 86%. Compound **7c** was taken to the next step without further purification.

*5-(3,4-Dichlorophenyl)-3-imino-2-(2-methoxy-3-pyridyl)amino-3,5-dihydrophenazine* (**7d**). Compound **7d** was synthesized in a similar manner as described for **7b** to afford 0.8 g of a red solid, yield 24%. Compound **7d** was taken to the next step without further purification.

### 3.6. General Procedure for Preparation of Compounds **8**–**25**

To a solution of **7a**–**d** (1.0 equiv.) and amine (2.0 equiv.) in dioxane was added glacial acetic acid (0.2 equiv.), then the mixture was stirred and heated at 110 °C in a sealed tube for 10–20 h. The mixture was concentrated under vacuum, and the residue was purified by column chromatography on silica gel, using petroleum ether/ethyl acetate to afford target products **8**–**25**.

*5-(3-Fluorophenyl)-3-isopropylimino-2-(2-methoxy-3-pyridyl)amino-3,5-dihydrophenazine* (**8**). Red solid. Yield 83 mg (37%). Mp: 198–199 °C. ^1^H-NMR (300 MHz, CDCl_3_) δ: 1.11 (6 H, brs), 3.42–3.50 (1 H, m), 4.04 (3 H, s), 5.29 (1 H, s), 6.47 (1 H, d, *J* = 8.1 Hz), 6.93 (2 H, brs), 7.10–7.20 (4 H, m), 7.37 (1 H, t, *J* = 8.1 Hz), 7.69–7.76 (2 H, m), 7.81–7.86 (2 H, m). ^13^C-NMR (100 MHz, CDCl_3_) δ: 23.5, 49.4, 53.7, 89.3, 100.1, 113.8, 116.5 (d, *J* = 22.3 Hz), 116.8, 117.0 (d, *J* = 20.6 Hz), 122.9, 124.8, 124.9, 127.6, 128.2, 131.4, 132.6, 132.7, 134.6, 135.5, 138.8, 138.9, 142.9, 150.6, 151.3, 155.5, 165.2 (d, *J* = 249.3 Hz). HRMS (ESI-TOF^+^): *m/z* [M + H]^+^ calcd for C_27_H_25_FN_5_O: 454.2038; found: 454.2043.

*5-(3-Fluorophenyl)-3-(4-tetrahydropyranyl)imino-2-(2-methoxy-3-pyridyl)amino-3,5-dihydro-phenazine* (**9**). Red solid. Yield 100 mg (41%). Mp: 190–191 °C. ^1^H-NMR (300 MHz, CDCl_3_) δ: 1.67 (4 H, brs), 3.41–3.50 (3 H, m), 4.01–4.04 (5 H, m), 5.26 (1 H, s), 6.50 (1 H, d, *J* = 8.1 Hz), 6.90–6.94 (1 H, m), 6.97 (1 H, s), 7.09–7.22 (4 H, m), 7.38 (1 H, t, *J* = 8.7 Hz), 7.69–7.76 (2 H, m), 7.81–7.86 (2 H, m), 9.06 (1 H, s). ^13^C-NMR (100 MHz, CDCl_3_) δ: 33.3, 33.4, 53.5, 53.7, 65.7, 89.0, 100.3, 113.9, 116.5 (d, *J* = 22.3 Hz), 116.8, 117.1(d, *J* = 20.6 Hz), 123.1, 124.4, 124.8, 127.8, 128.3, 131.2, 132.6, 132.7, 134.8, 135.6, 138.7, 138.9, 142.6, 150.9, 151.2, 155.3, 165.2 (d, *J* = 249.3 Hz). HRMS (ESI-TOF^+^): *m/z* [M + H]^+^ calcd for C_29_H_27_FN_5_O_2_: 496.2143; found: 496.2152.

*5-(3-Chlorophenyl)-3-isopropylimino-2-(2-methoxy-3-pyridyl)amino-3,5-dihydrophenazine* (**10**). Red solid. Yield 110 mg (33%). Mp: 215–217 °C. ^1^H-NMR (300 MHz, CDCl_3_) δ: 1.11 (6 H, d, *J* = 6.0 Hz), 3.46 (1 H, m), 4.04 (3 H, s), 5.27 (1 H, s), 6.45 (1 H, m), 6.91 (2 H, m), 7.15 (2 H, m), 7.27 (1 H, m), 7.38 (1 H, s), 7.64 (2 H, m), 7.68 (1 H, m), 7.83 (2 H, m), 8.90 (1 H, brs). ^13^C-NMR (100 MHz, CDCl_3_) δ: 23.5, 49.4, 53.7, 89.4, 100.1, 113.8, 116.8, 122.9, 124.8, 124.9, 127.3, 127.6, 128.2, 129.4, 130.1, 131.4, 132.3, 134.6, 135.5, 136.7, 138.7, 138.8, 142.9, 150.6, 151.2, 155.5. HRMS (ESI-TOF^+^): *m/z* [M + H]^+^ calcd for C_27_H_25_ClN_5_O: 470.1712; found: 470.1696.

*5-(3-Chlorophenyl)-3-cyclopropylimino-2-(2-methoxy-3-pyridyl)amino-3,5-dihydrophenazine* (**11**). Red solid. Yield 149 mg (46%). Mp: 211–214 °C. ^1^H-NMR (300 MHz, CDCl_3_) δ: 0.84 (4 H, m), 2.72 (1 H, m), 4.03 (3 H, s), 5.54 (1 H, s),6.44 (1 H, m), 6.90 (2 H, m), 7.17 (3 H, m), 7.38 (1 H, s), 7.66 (2 H, m), 7.74 (1 H, m), 7.80 (2 H, m). ^13^C-NMR (100 MHz, CDCl_3_) δ: 10.1, 32.9, 53.7, 89.8, 100.2, 113.9, 116.8, 124.4, 124.7, 127.1, 127.2, 127.8, 128.3, 129.3, 130.1, 132.3, 134.6, 136.7, 138.6, 138.9, 142.2, 142.7, 150.9, 153.5, 155.4, 161.0. HRMS (ESI-TOF^+^): *m/z* [M + H]^+^ calcd for C_27_H_23_ClN_5_O: 468.1586; found: 468.1573.

*5-(3-Chlorophenyl)-3-cyclobutylimino-2-(2-methoxy-3-pyridyl)amino-3,5-dihydrophenazine* (**12**). Red solid. Yield 142 mg (42%). Mp: 205–208 °C. ^1^H-NMR (300 MHz, CDCl_3_) δ: 1.75 (2 H, m), 2.07 (2 H, m), 2.20 (2 H, m), 3.93 (1 H, m), 4.04 (3 H, s), 5.08 (1 H, s), 6.50 (1 H, m), 6.93 (2 H, m), 7.17 (3 H, m), 7.38 (1 H, s), 7.69 (3 H, m), 7.84 (2 H, m). ^13^C-NMR (100 MHz, CDCl_3_) δ: 16.1, 32.0, 53.7, 54.9, 90.9, 100.3, 113.9, 116.8, 123.1, 124.8, 127.4, 127.8, 128.3, 129.4, 130.1, 131.2, 132.2, 134.2, 135.6, 136.7, 138.7, 138.9, 142.6, 142.8, 151.2, 151.4, 155.4. HRMS (ESI-TOF^+^): *m/z* [M + H]^+^ calcd for C_28_H_25_ClN_5_O: 482.1742; found: 482.1703.

*5-(3-Chlorophenyl)-3-cyclohexylimino-2-(2-methoxy-3-pyridyl)amino-3,5-dihydrophenazine* (**13**). Red solid. Yield 62 mg (49%). Mp: 197–201 °C.^1^H-NMR (300 MHz, CDCl_3_) δ: 1.10 (3 H, m), 1.28 (2 H, m), 1.53 (3 H, m), 1.68 (2 H, m), 2.98 (1 H, m), 3.94 (3 H, s), 5.12 (1 H, s), 6.55 (2 H, m), 6.81 (1 H, s), 7.11 (3 H, m), 7.55 (2 H, m), 7.81 (5 H, m), 9.00 (1 H, s). ^13^C-NMR (100 MHz, CDCl_3_) δ: 23.8, 25.4, 33.0, 53.6, 56.9, 88.7, 100.1, 113.9, 117.4, 122.8, 124.0, 124.1, 127.8, 127.9, 128.0, 128.2, 129.0, 130.0, 131.0, 132.9, 134.2, 135.1, 135.3, 138.2, 138.5, 141.5, 150.1, 150.3. HRMS (ESI-TOF^+^): *m/z* [M + H]^+^ calcd for C_30_H_29_ClN_5_O: 510.2061; found: 510.2059.

*5-(3-Chlorophenyl)-3-(4-tetrahydropyranyl)imino-2-(2-methoxy-3-pyridyl)amino-3,5-dihydrophenazine* (**14**). Red solid. Yield 308 mg (86%). Mp: 203–206 °C. ^1^H-NMR (300 MHz, CDCl_3_) δ: 1.75 (4 H, m), 2.85 (1 H, m), 3.43 (2 H, m), 3.96 (2 H, m), 4.04 (3 H, s), 5.27 (1 H, s), 6.53 (1 H, m), 6.90 (2 H, m), 7.17 (3 H, m), 7.38 (1 H, s), 7.64 (2 H, m), 7.74 (1 H, m), 7.84 (2 H, m), 9.08 (1 H, s). ^13^C-NMR (100 MHz, CDCl_3_) δ: 34.2, 53.5, 53.8, 66.9, 89.1, 100.4, 113.9, 116.7, 124.4, 124.7, 127.1, 127.2, 127.8, 128.3, 129.3, 130.1, 132.3, 134.6, 136.7, 138.6, 138.9, 142.2, 142.7, 150.9, 153.5, 155.4, 161.0. HRMS (ESI-TOF^+^): *m/z* [M + H]^+^ calcd for C_29_H_27_ClN_5_O_2_: 512.1848; found: 512.1838.

*5-(3,4-Difluorophenyl)-3-isopropylimino-2-(2-methoxy-3-pyridyl)amino-3,5-dihydrophenazine* (**15**). Red solid. Yield 270 mg (80%). Mp: 229–231 °C. ^1^H-NMR (300 MHz, CDCl_3_) δ: 1.13 (6 H, d, *J* = 6.9 Hz), 3.45–3.53 (1 H, m, *J* = 6.3 Hz), 4.04 (3 H, s), 5.28 (1 H, s), 6.43 (1 H, d, *J* = 7.5 Hz), 6.89–6.93 (2 H, m), 7.13–7.25 (4 H, m), 7.49–7.59 (1 H, m), 7.68 (1 H, d, *J* = 7.2 Hz), 7.81–7.84 (2 H, m), 8.92 (1 H, s). ^13^C-NMR (100 MHz, CDCl_3_) δ: 23.5, 23.6, 49.5, 53.7, 89.2, 100.1, 113.5, 116.8, 118.8 (d, *J* = 17.8 Hz), 120.0 (d, *J* = 18.5 Hz), 123.1, 124.8, 124.9, 125.8, 127.7, 128.3, 131.4, 133.7, 134.7, 135.5, 138.9, 142.9, 151.0 (dd, *J* = 251.8, 12.0 Hz), 151.2, 152.0 (dd, *J* = 252.6, 13.2 Hz), 155.5. HRMS (ESI-TOF^+^): *m/z* [M + H]^+^ calcd for C_27_H_24_F_2_N_5_O: 472.1943; found: 472.1937.

*5-(3,4-Difluorophenyl)-3-cyclopropylimino-2-(2-methoxy-3-pyridyl)amino-3,5-dihydrophenazine* (**16**). Red solid. Yield 233 mg (yield 71%). Mp: 213–214 °C. ^1^H-NMR (300 MHz, CDCl_3_) δ: 0.86 (2 H, d, *J* = 2.7 Hz), 0.93 (2 H, d, *J* = 6.0 Hz), 2.77 (1 H, m), 4.01 (3 H, s), 5.54 (1 H, s), 6.42 (1 H, d, *J* = 8.4 Hz), 6.86 (1 H, s), 6.99 (1 H, m), 7.12 (4 H, m), 7.52 (1 H, m), 7.66 (1 H, d, *J* = 8.1 Hz), 7.82 (2 H, m), 8.58 (1 H, s). ^13^C-NMR (100 MHz, CDCl_3_): δ: 10.2, 33.0, 53.7, 89.7, 100.1, 113.5, 116.8, 118.9 (d, *J* = 17.9 Hz), 120.0 (d, *J* = 18.3 Hz), 123.1, 124.7, 125.1, 125.9, 127.7, 128.2, 131.6, 133.7, 134.5, 135.7, 139.0, 142.7, 151.0 (dd, *J* = 254.5, 13.1 Hz), 151.4, 151.9 (dd, *J* = 252.9, 13.5 Hz), 155.5. HRMS (ESI-TOF^+^): *m/z* [M + H]^+^ calcd for C_27_H_22_F_2_N_5_O: 470.1787; found: 470.1762.

*5-(3,4-Difluorophenyl)-3-cyclobutylimino-2-(2-methoxy-3-pyridyl)amino-3,5-dihydrophenazine* (**17**). Red solid. Yield 300 mg (78%). Mp: 200–201 °C. ^1^H-NMR (300 MHz, CDCl_3_) δ: 1.70–1.82 (2 H, m), 2.03–2.20 (4 H, m), 3.81–4.04 (1 H, m), 4.04 (3 H, s), 5.08 (1 H, s), 6.46 (1 H, d, *J* = 7.5 Hz), 6.89–6.93 (2 H, m), 7.12–7.23 (4 H, m), 7.50–7.59 (1 H, m), 7.69 (1 H, d, *J* = 7.5 Hz), 7.82–7.84 (2 H, m). ^13^C-NMR (100 MHz, CDCl_3_) δ: 16.1, 32.0, 53.7, 54.9, 90.7, 100.3, 113.6, 116.8, 118.8 (d, *J* = 17.7 Hz), 119.9 (d, *J* = 18.1 Hz), 123.2, 124.7, 124.9, 125.8, 127.8, 128.4, 131.3, 133.6, 134.2, 135.6, 138.9, 142.7, 151.0 (dd, *J* = 251.1, 11.8 Hz), 151.1, 151.3, 151.9 (dd, *J* = 253.4, 13.7 Hz), 155.4. HRMS (ESI-TOF^+^): *m/z* [M + H]^+^ calcd for C_28_H_24_F_2_N_5_O: 484.1943; found: 484.1933.

*5-(3,4-Difluorophenyl)-3-cyclohexylimino-2-(2-methoxy-3-pyridyl)amino-3,5-dihydrophenazine* (**18**). Red solid. Yield 330 mg (81%). Mp: 218–219 °C. ^1^H-NMR (300 MHz, CDCl_3_) δ: 1.23–1.79 (10 H, m), 3.14–3.20 (1 H, m), 4.03 (3 H, s), 5.26 (1 H, s), 6.45 (1 H, d, *J* = 7.5 Hz), 6.88–6.93 (2 H, m), 7.11–7.25 (4 H, m), 7.49–7.58 (1 H, m), 7.69 (1 H, d, *J* = 7.5 Hz), 7.80–7.84 (2 H, m). ^13^C-NMR (100 MHz, CDCl_3_) δ: 24.2, 25.9, 33.5, 33.6, 53.7, 57.4, 89.4, 100.1, 113.5, 116.8, 118.8 (d, *J* = 17.8 Hz), 119.8 (d, *J* = 18.1 Hz), 123.0, 124.5, 124.9, 125.8, 127.7, 128.3, 131.4, 133.7, 134.6, 135.6, 138.7, 142.8, 150.4, 151.0 (dd, *J* = 251.5, 11.9 Hz), 151.3, 151.9 (dd, *J* = 252.6, 13.5 Hz), 155.4. HRMS (ESI-TOF^+^): *m/z* [M + H]^+^ calcd for C_30_H_28_F_2_N_5_O: 512.2256; found: 512.2263.

*5-(3,4-Difluorophenyl)-3-(4-tetrahydropyranyl)imino-2-(2-methoxy-3-pyridyl)amino-3,5-dihydro-phenazine* (**19**). Red solid. Yield 240 mg (59%). Mp: 255–256 °C. ^1^H-NMR (300 MHz, CDCl_3_) δ: 1.65–1.68 (4 H, m), 3.46–3.55 (3 H, m), 4.05 (5 H, m), 5.25 (1 H, s), 6.46 (1 H, d, *J* = 9.0 Hz), 6.90–6.95 (2 H, m), 7.13–7.24 (4 H, m), 7.51–7.60 (1 H, m), 7.71 (1 H, d, *J* = 7.5 Hz), 7.81–7.85 (2 H, m), 9.08 (1 H, s).^13^C-NMR (100 MHz, CDCl_3_) δ: 33.2, 33.4, 53.3, 53.8, 65.5, 88.9, 100.3, 113.7, 116.8, 118.7 (d, *J* = 19.7 Hz), 119.9 (d, *J* = 18.1 Hz), 123.3, 124.3, 124.8, 125.7, 127.9, 128.4, 131.2, 133.5, 134.9, 135.6, 138.8, 142.8, 150.9, 151.0 (dd, *J* = 251.4, 13.6 Hz), 151.9 (dd, *J* = 252.8, 13.4 Hz), 155.3. HRMS (ESI-TOF^+^): *m/z* [M + H]^+^ calcd for C_29_H_26_F_2_N_5_O_2_: 514.2049; found: 514.2042.

*5-(3,4-Difluorophenyl)-3-(4-methoxycyclohexyl)imino-2-(2-methoxy-3-pyridyl)amino-3,5-dihydro-phenazine* (**20**). Red solid. Yield 432 mg (84%). Mp: 214–215 °C. ^1^H-NMR (300 MHz, CDCl_3_) δ: 1.26 (2 H, m), 1.40 (2 H, m), 1.73 (2 H, m), 2.05 (2 H, m), 3.16 (1 H, m), 3.27 (1 H, m), 3.36 (3 H, s), 4.03 (3 H, s), 5.26 (1 H, s), 6.46 (1 H, d, *J* = 7.5 Hz), 6.92 (2 H, m), 7.15 (3 H, m), 7.22 (1 H, m), 7.53 (1 H, m), 7.69 (1 H, d, *J* = 8.1 Hz), 7.82 (2 H, m), 8.94 (1 H, brs). ^13^C-NMR (100 MHz, CDCl_3_) δ: 29.3, 29.5, 30.8, 53.7, 55.8, 56.9, 78.2, 89.2, 100.2, 113.6, 116.8, 118.7 (d, *J* = 18.0 Hz), 119.9 (d, *J* = 18.9 Hz), 123.2, 124.7, 125.7, 127.8, 128.4, 131.4, 133.5, 134.7, 135.6, 138.8, 142.7, 151.0 (dd, *J* = 252.5, 13.7 Hz), 151.1, 151.9 (dd, *J* = 252.2, 13.5 Hz), 155.4. HRMS (ESI-TOF^+^): *m/z* [M + H]^+^ calcd for C_31_H_30_F_2_N_5_O_2_: 542.2362; found: 542.2338.

*5-(3,4-Dichlorophenyl)-3-isopropylimino-2-(2-methoxy-3-pyridyl)amino-3,5-dihydrophenazine* (**21**). Red solid. Yield 130 mg (68%). Mp: 245–247 °C. ^1^H-NMR (300 MHz, CDCl_3_) δ: 1.13 (6 H, d, *J* = 6.3 Hz), 3.50 (1 H, m), 4.03 (3 H, s), 5.29 (1 H, s), 6.43 (1 H, d, *J* = 6.3 Hz), 6.89 (1 H, s), 6.93 (1 H, m), 7.10–7.23 (3 H, m), 7.49 (1 H, d, *J* = 2.4 Hz), 7.68 (1 H, m), 7.80–7.84 (3 H, m). ^13^C-NMR (100 MHz, CDCl_3_) δ: 23.6, 49.5, 53.7, 89.3, 100.1, 113.6, 116.8, 123.1, 124.8, 125.0, 127.7, 128.3, 128.6, 131.3, 133.1, 134.3, 134.5, 135.3, 135.5, 136.8, 138.9, 142.9, 150.4, 151.2, 155.5. HRMS (ESI-TOF^+^): *m/z* [M + H]^+^ calcd for C_27_H_24_Cl_2_N_5_O: 504.1853; found: 504.1852.

*5-(3,4-Dichlorophenyl)-3-cyclopropylimino-2-(2-methoxy-3-pyridyl)amino-3,5-dihydrophenazine* (**22**). Red solid. Yield 120 mg (65%). Mp: 215–217 °C. ^1^H-NMR (300 MHz, CDCl_3_) δ: 0.86–0.96 (4 H, m), 2.80 (1 H, m), 4.00 (3 H, s), 5.54 (1 H, s), 6.41 (1 H, d, *J* = 8.7 Hz), 6.86 (1 H, s), 6.91 (1 H, m), 7.10–7.28 (3 H, m), 7.51 (1 H, d, *J* = 2.1 Hz), 7.65 (1 H, m), 7.80–7.83 (3 H, m). ^13^C-NMR (100 MHz, CDCl_3_) δ: 10.3, 33.1, 53.7, 89.8, 100.0, 113.6, 116.8, 123.1, 124.6, 125.2, 127.7, 128.2, 128.8, 131.4, 133.2, 134.3, 135.4, 135.7, 136.8, 139.0, 142.7, 151.4, 152.2, 155.4. HRMS (ESI-TOF^+^): *m/z* [M + H]^+^ calcd for C_27_H_22_Cl_2_N_5_O: 502.1317; found: 502.1316.

*5-(3,4-Dichlorophenyl)-3-cyclobutylimino-2-(2-methoxy-3-pyridyl)amino-3,5-dihydrophenazine* (**23**). Red solid. Yield 167 mg (65%). Mp: 216–219 °C. ^1^H-NMR (300 MHz, CDCl_3_) δ: 1.81 (2 H, m), 2.08 (2 H, m), 2.22 (2 H, m), 3.95 (1 H, m), 4.05 (3 H, s), 5.11 (1 H, s), 6.47 (1 H, d, *J* = 7.5 Hz), 6.91 (2 H, m), 7.17 (2 H, m), 7.23 (1 H, m), 7.50 (1 H, d, *J* = 2.1 Hz), 7.69 (1 H, d, *J* = 7.5 Hz), 7.84 (3 H, m), 8.79 (1 H, brs). ^13^C-NMR (100 MHz, CDCl_3_) δ: 16.1, 32.0, 53.7, 54.8, 90.8, 100.3, 113.7, 116.8, 123.2, 124.7, 124.9, 127.8, 128.4, 128.6, 131.1, 131.3, 133.0, 134.0, 134.3, 135.3, 135.6, 136.7, 139.0, 142.8, 151.1, 151.2, 155.4. HRMS (ESI-TOF^+^): *m/z* [M + H]^+^ calcd for C_28_H_24_Cl_2_N_5_O: 516.1352; found: 516.1322.

*5-(3,4-Dichlorophenyl)-3-(4-tetrahydropyranyl)imino-2-(2-methoxy-3-pyridyl)amino-3,5-dihydro-phenazine* (**24**). Red solid. Yield 140 mg (67%). Mp: 232–233 °C. ^1^H-NMR (300 MHz, CDCl_3_) δ: 1.61–1.72 (4 H, m), 3.48–3.52 (3 H, m), 4.04 (5 H, m), 5.26 (1 H, s), 6.47 (1 H, d, *J* = 7.8 Hz), 6.90–6.94 (1 H, m), 6.95 (1 H, s), 7.13–7.23 (3 H, m), 7.49 (1 H, s), 7.70 (1 H, d, *J* = 7.2 Hz), 7.81–7.85 (3 H, m). ^13^C-NMR (100 MHz, CDCl_3_) δ: 33.3, 53.3, 53.8, 65.5, 89.1, 100.3, 113.7, 116.8, 123.3, 124.4, 124.8, 127.9, 128.4, 128.5, 131.0, 131.2, 133.1, 134.4, 134.7, 135.4, 135.6, 136.7, 138.8, 142.6, 150.9, 155.3. HRMS (ESI-TOF^+^): *m/z* [M + H]^+^ calcd for C_29_H_26_Cl_2_N_5_O_2_: 546.1875; found: 546.1874.

*5-(3,4-Dichlorophenyl)-3-(4-methoxycyclohexyl)imino-2-(2-methoxy-3-pyridyl)amino-3,5-dihydro-phenazine* (**25**). Red solid. Yield 100 mg (45%). Mp: 215–217 °C. ^1^H-NMR (300 MHz, CDCl_3_) δ: 1.23–1.51 (4 H, m), 1.73–2.10 (4 H, m), 3.15–3.29 (2 H, m), 3.37 (3 H, s), 3.78 (3 H, s), 5.27 (1 H, s), 6.48 (1 H, d, *J* = 8.4 Hz), 6.90–6.93 (2 H, m), 7.12–7.24 (3 H, m), 7.49 (1 H, d, *J* = 2.1 Hz), 7.70 (1 H, d, *J* = 7.2 Hz), 7.79–7.84 (3 H, m). ^13^C-NMR (100 MHz, CDCl_3_) δ: 29.3, 29.4, 30.7, 53.7, 55.8, 56.8, 89.3, 100.2, 113.7, 116.8, 123.2, 124.7, 124.8, 127.8, 128.4, 128.5, 131.1, 131.2, 133.0, 134.4, 134.5, 135.3, 135.6, 136.7, 138.8, 142.7, 151.0, 151.1, 155.4. HRMS (ESI-TOF^+^): *m/z* [M + H]^+^ calcd for C_31_H_30_Cl_2_N_5_O_2_: 574.2129; found: 574.2128.

## 4. Conclusions

A systematic structure-activity relationship study was conducted based on riminophenazine pharmacophore containing a 2-methoxypyridylamino group at the C-2 position. This side chain was identified as having potent antituberculosis activity and less lipophilicity in our previous study. Although the number, type and position of the halogen atoms on the phenyl ring at the N-5 position have no significant impact on the antituberculosis activity, the halogen substitution pattern appears important for cytotoxicity. The 3,4-dihalogen substitution pattern displayed significantly lower cytotoxicity. Compound **15** exhibited good pharmacokinetic properties, low skin pigmentation potential and excellent efficacy *in vivo* and warrants further evaluation. Our observations indicate that novel riminophenazine derivatives with a 2-methoxypyridylamino moiety as the promising pharmacophore have the potential to deliver a new drug candidate for the treatment of TB and MDR-TB.

## Supplementary Materials

Supplementary materials can be accessed at: http://www.mdpi.com/1420-3049/19/4380/s1.
