# Supplementary material for: Synthesis and Biological Evaluation of Novel 2-Methoxypyridylamino-Substituted Riminophenazine Derivatives as Antituberculosis Agents
Source: Molecules. 2014 Apr 9;19(4):4380–94. doi: 10.3390/molecules19044380 (PMC6271099; doi:10.3390/molecules19044380)

Supplementary Materials

^{1}H and ^{13}C-NMR of compounds **8**–**25**

5-(3-Fluorophenyl)-3-isopropylimino-2-(2-methoxy-3-pyridyl)amino-3,5-dihydrophenazine (**8**)

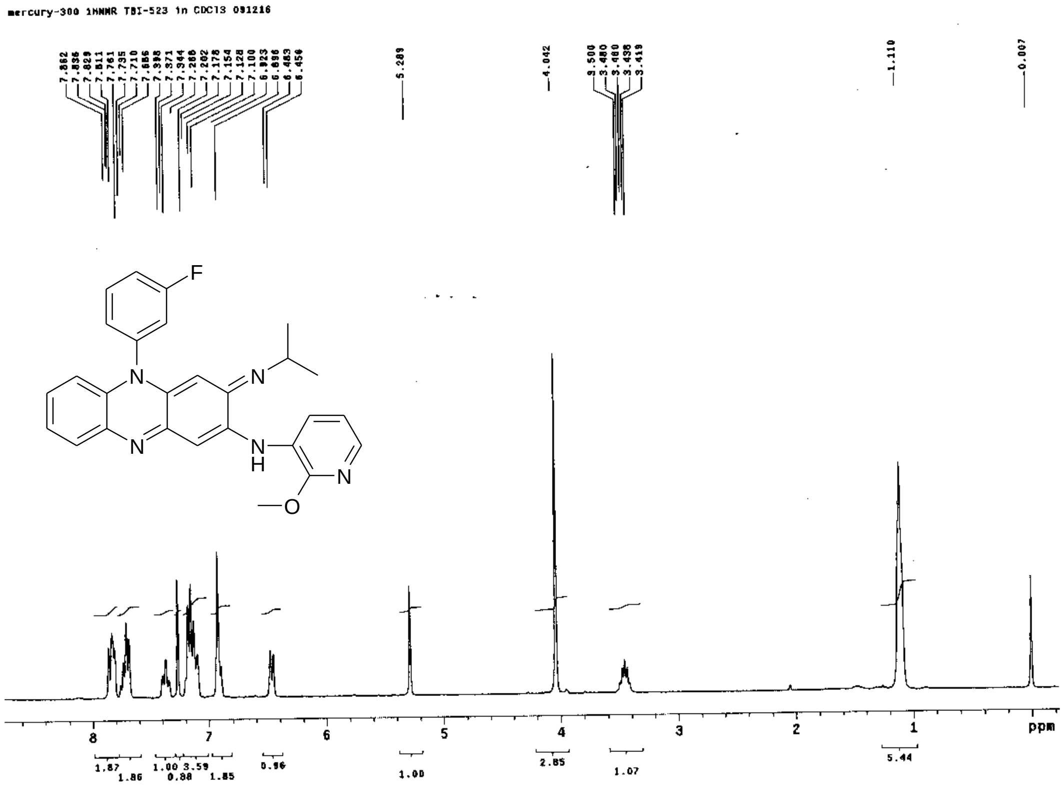

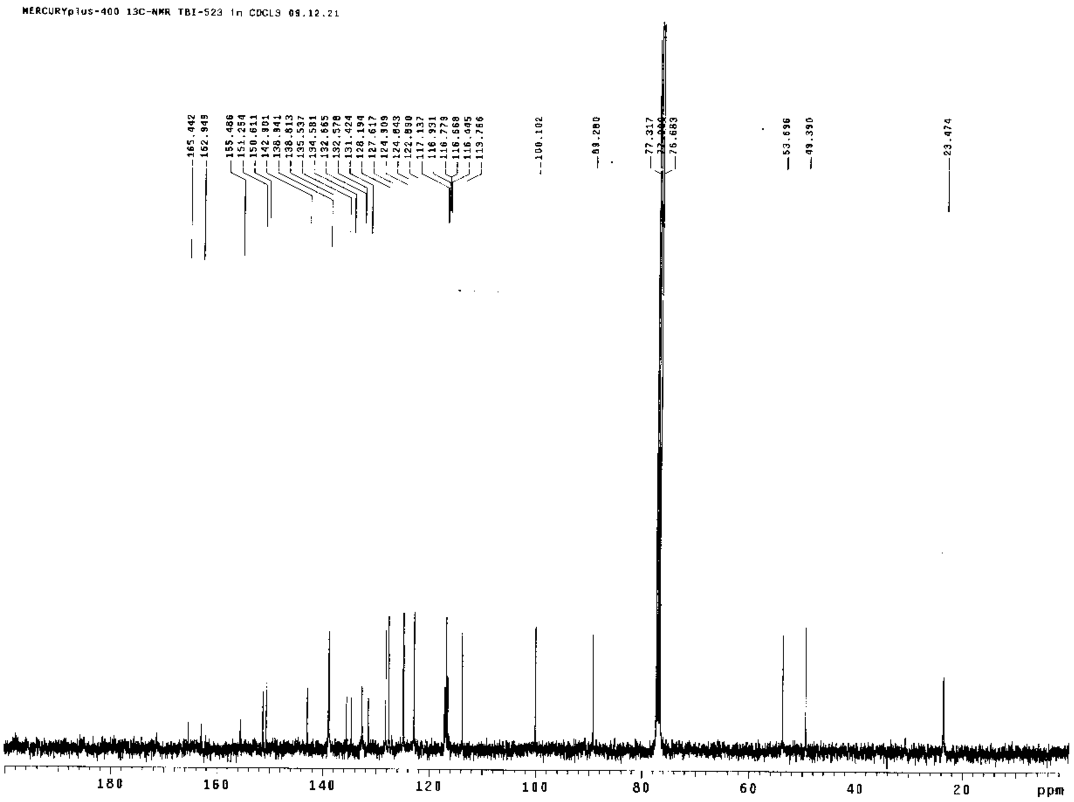

5-(3-Fluorophenyl)-3-(4-tetrahydropyranyl)imino-2-(2-methoxy-3-pyridyl)amino-3,5-dihydrophenazine (**9**)

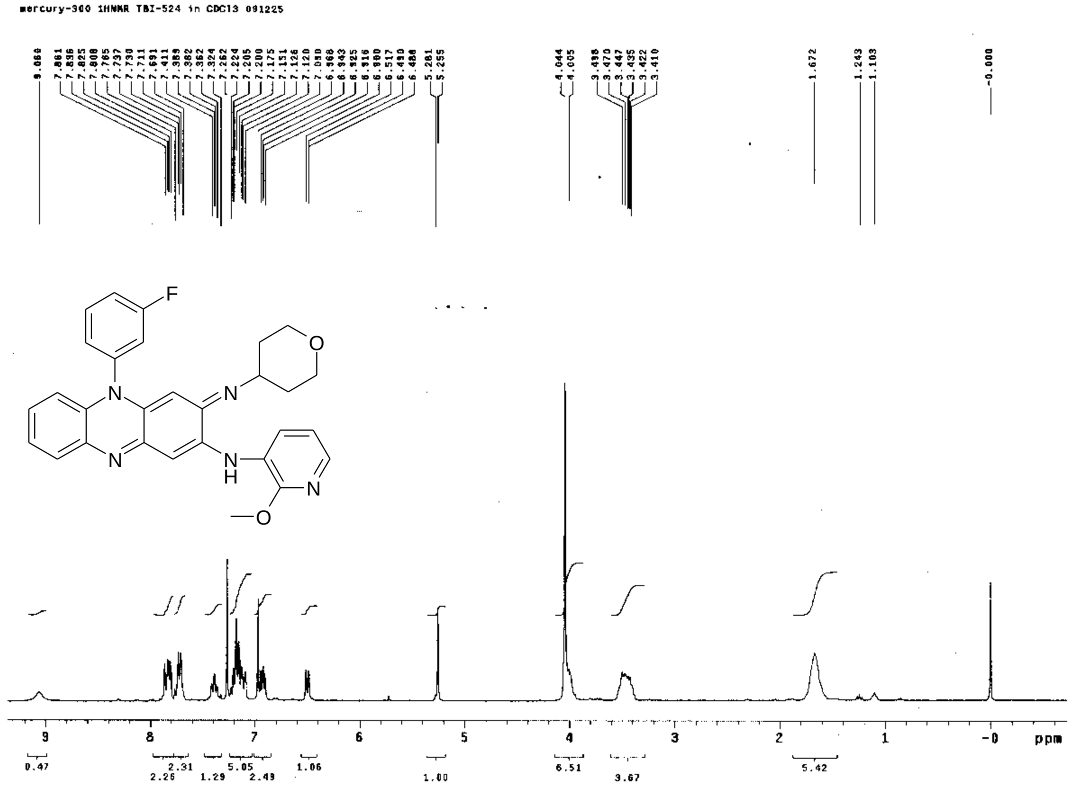

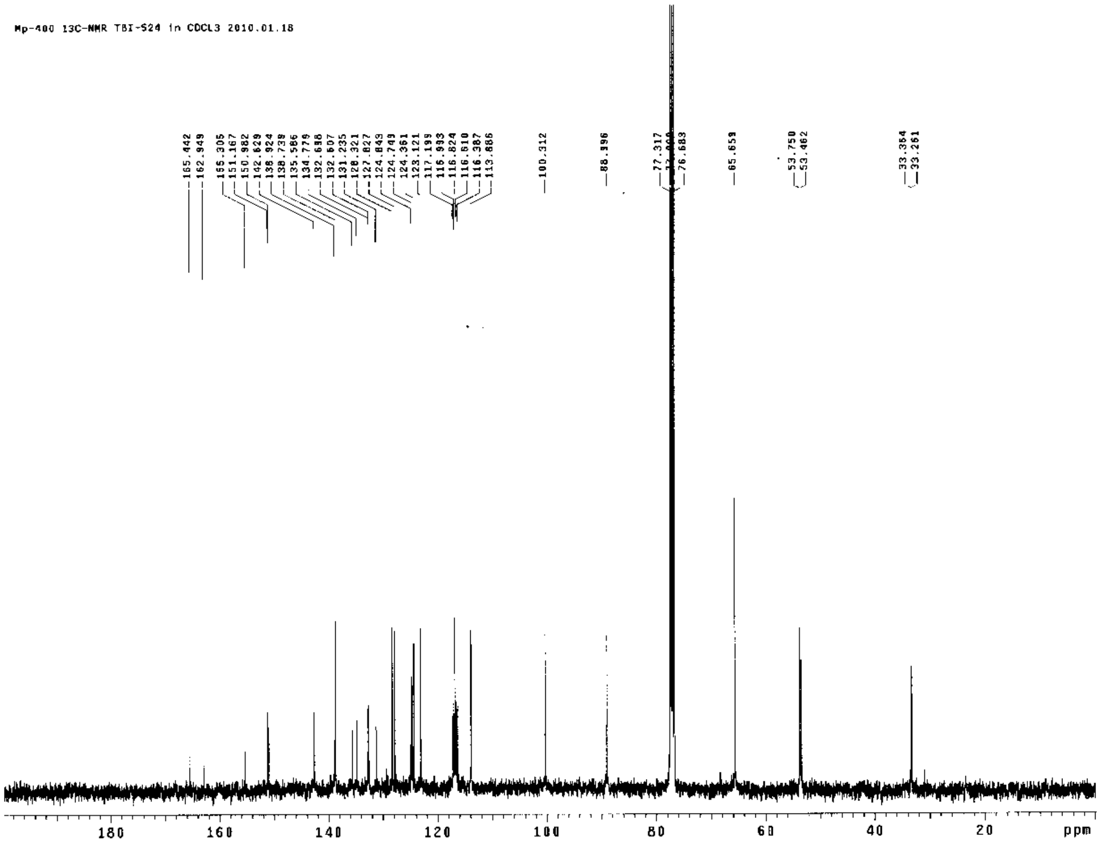

5-(3-Chlorophenyl)-3-isopropylimino-2-(2-methoxy-3-pyridyl)amino-3,5-dihydrophenazine (**10**)

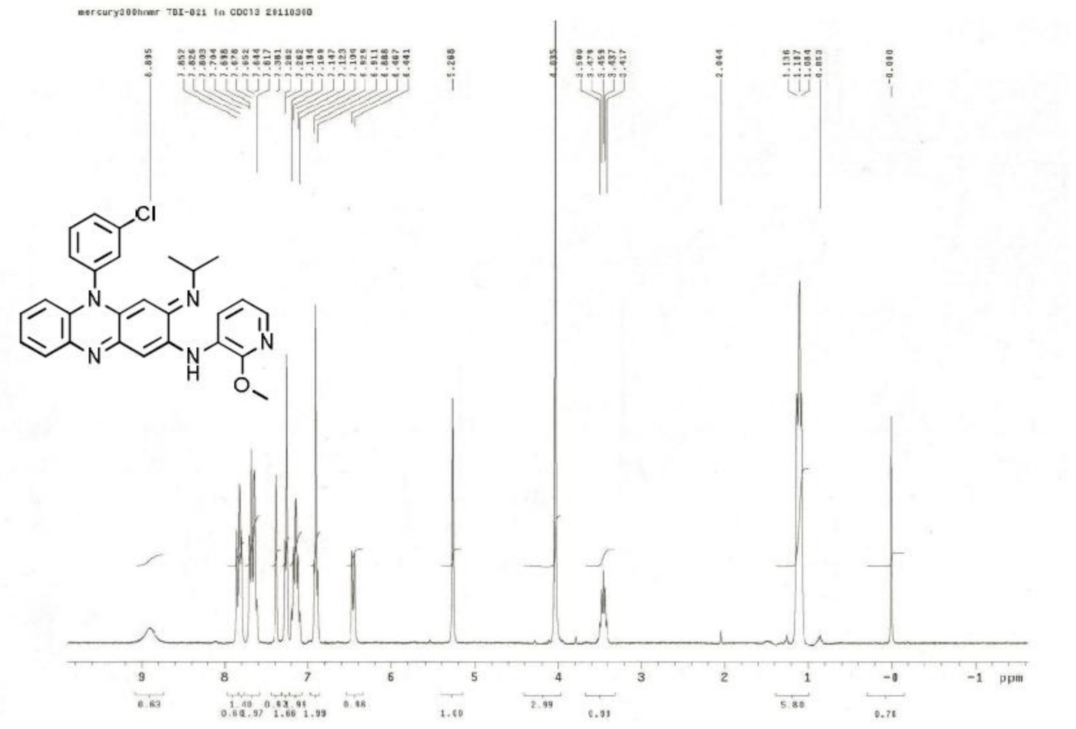

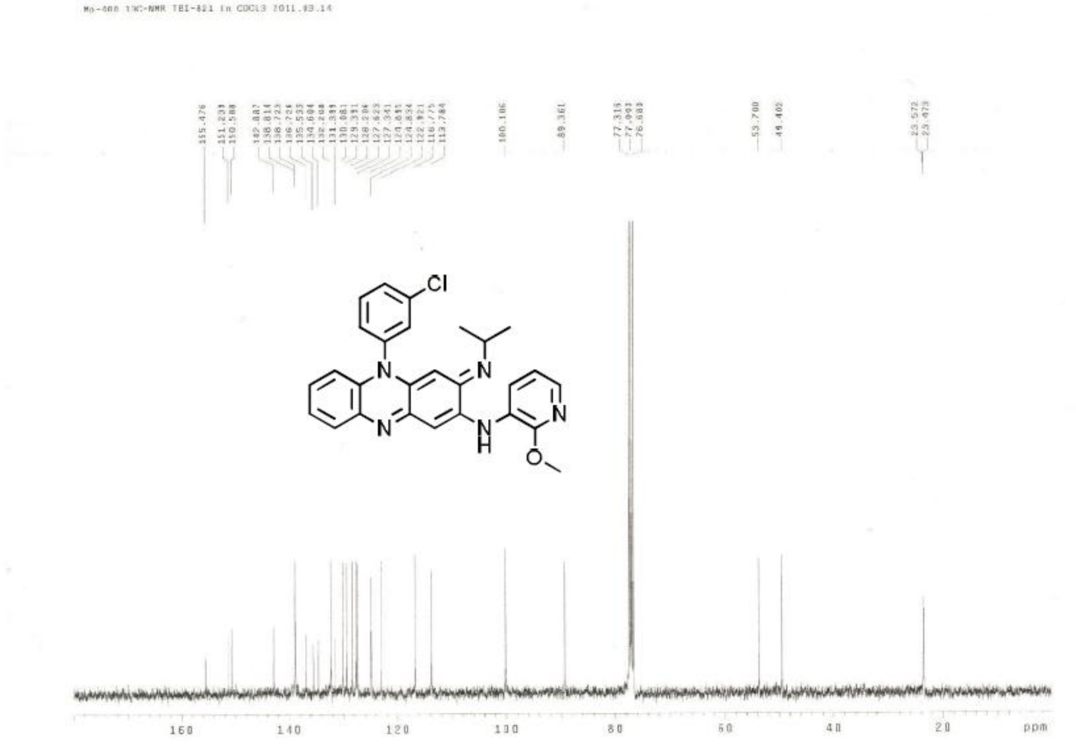

5-(3-Chlorophenyl)-3-cyclopropylimino-2-(2-methoxy-3-pyridyl)amino-3,5-dihydrophenazine (**11**)

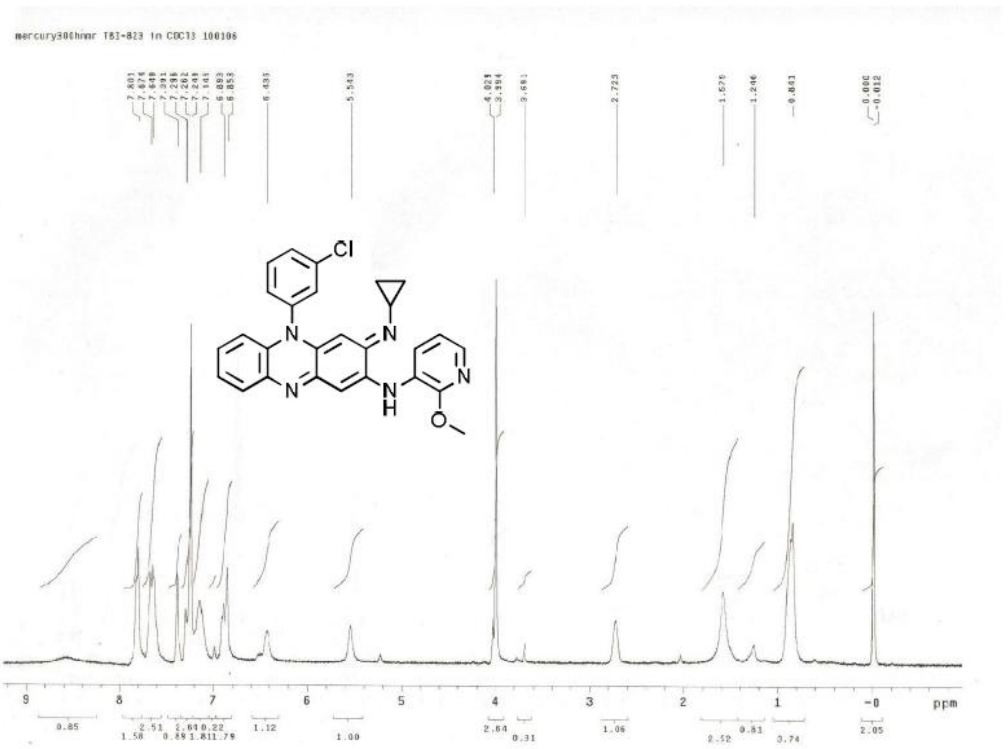

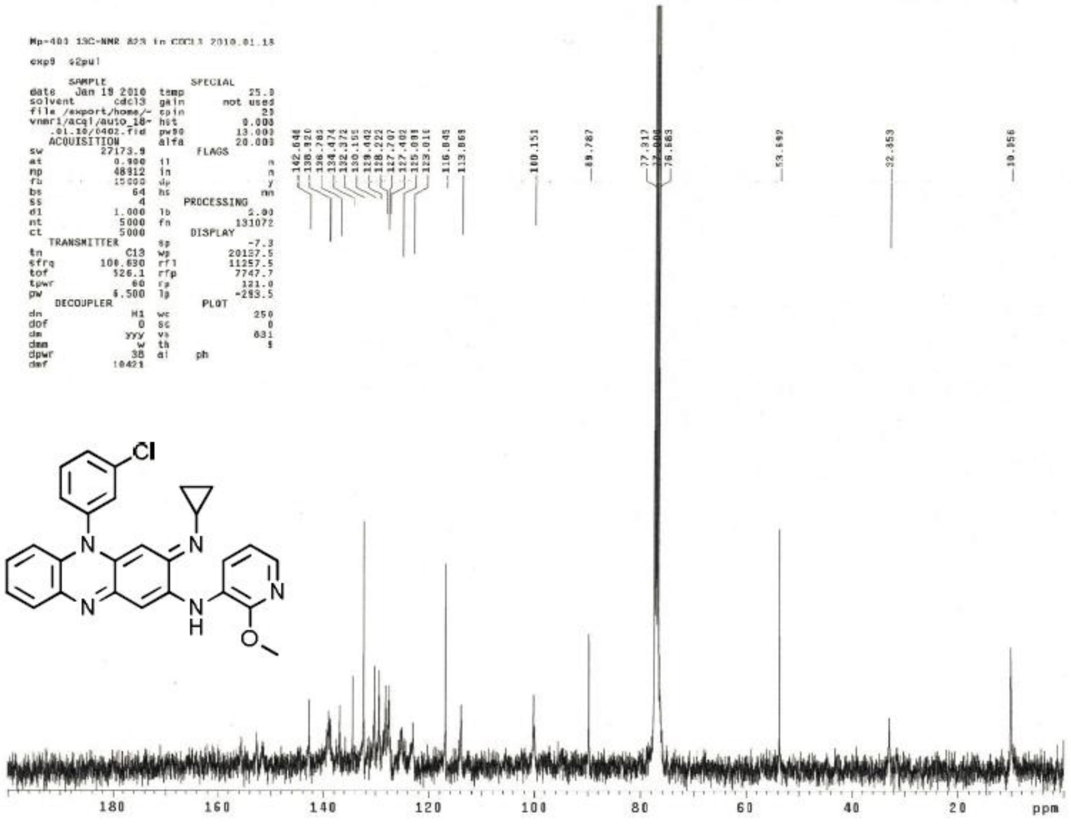

5-(3-Chlorophenyl)-3-cyclobutylimino-2-(2-methoxy-3-pyridyl)amino-3,5-dihydrophenazine (**12**)

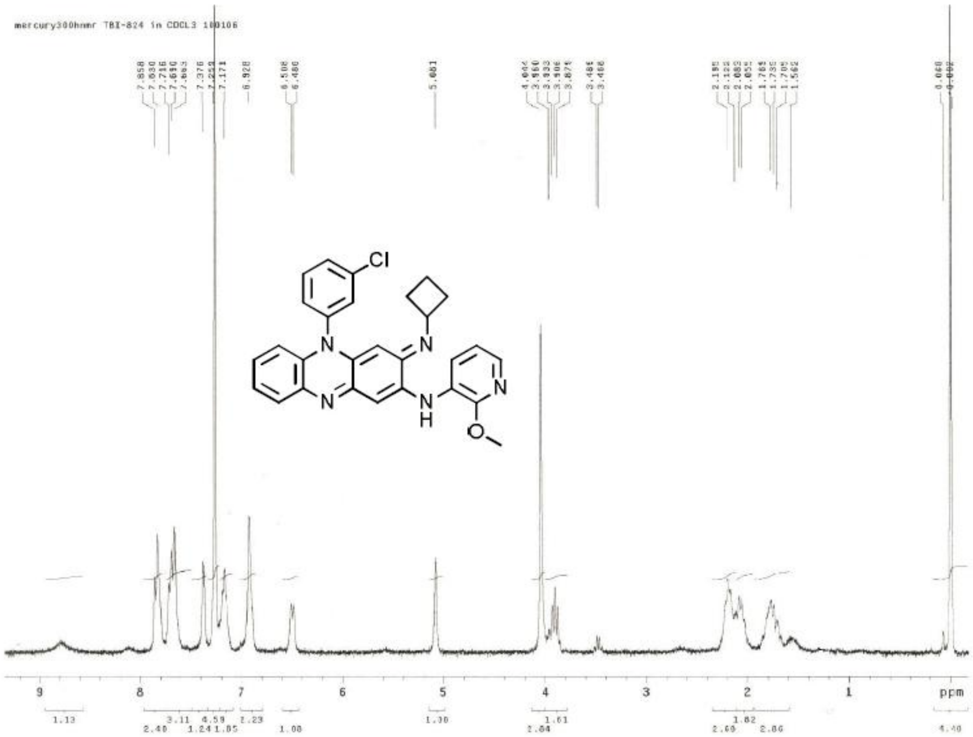

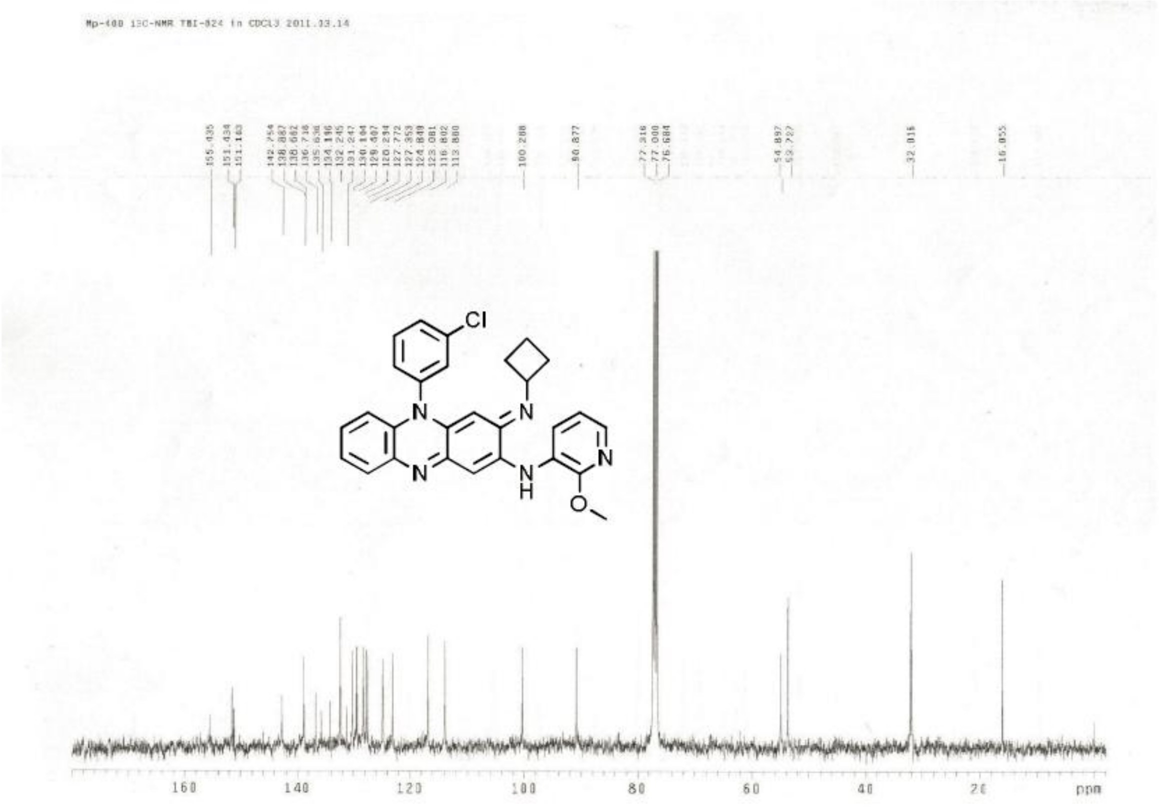

5-(3-Chlorophenyl)-3-cyclohexylimino-2-(2-methoxy-3-pyridyl)amino-3,5-dihydrophenazine (**13**)

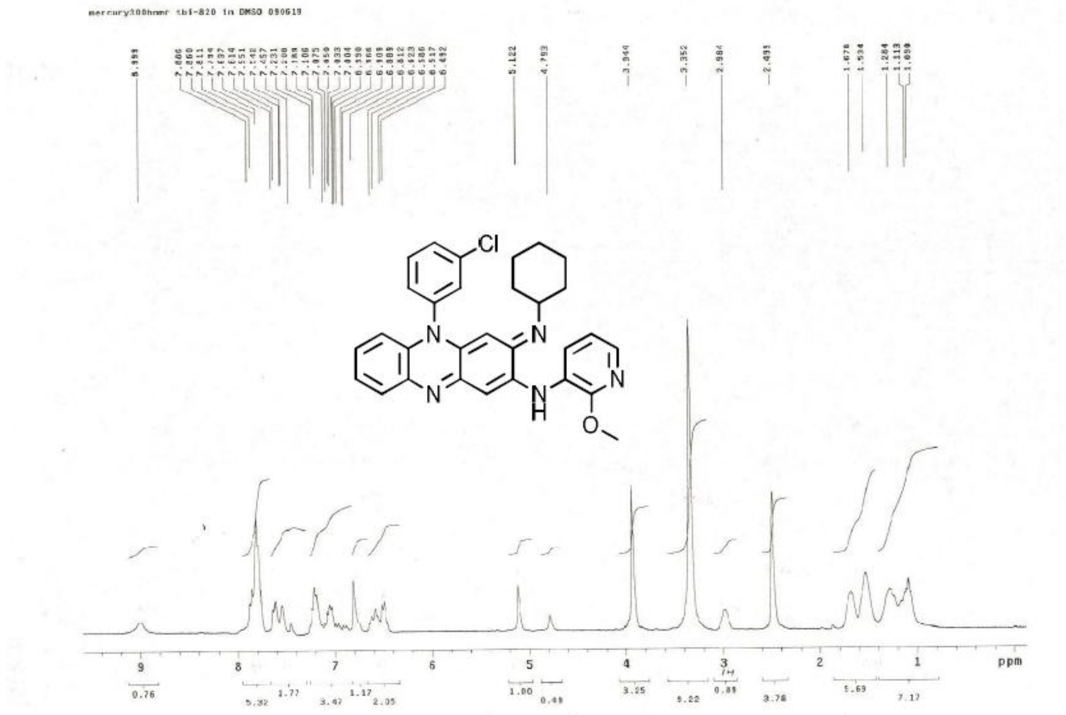

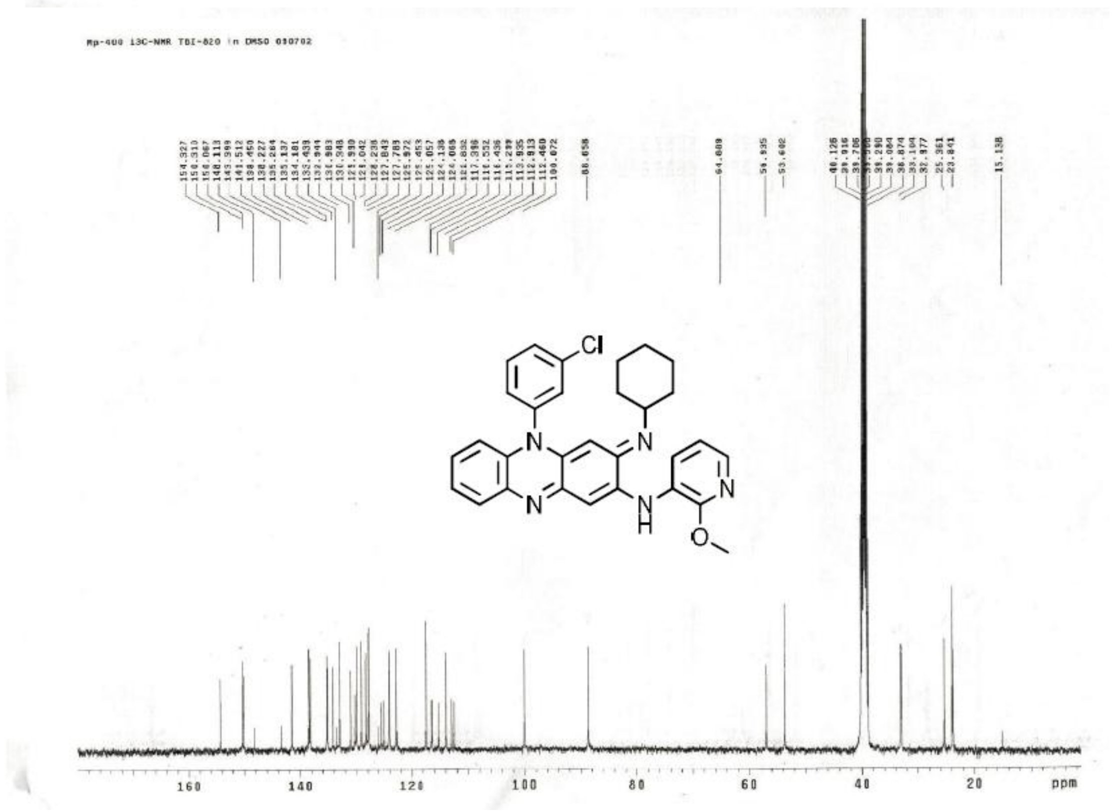

5-(3-Chlorophenyl)-3-(4-tetrahydropyranyl)imino-2-(2-methoxy-3-pyridyl)amino-3,5-dihydrophenazine (**14**)

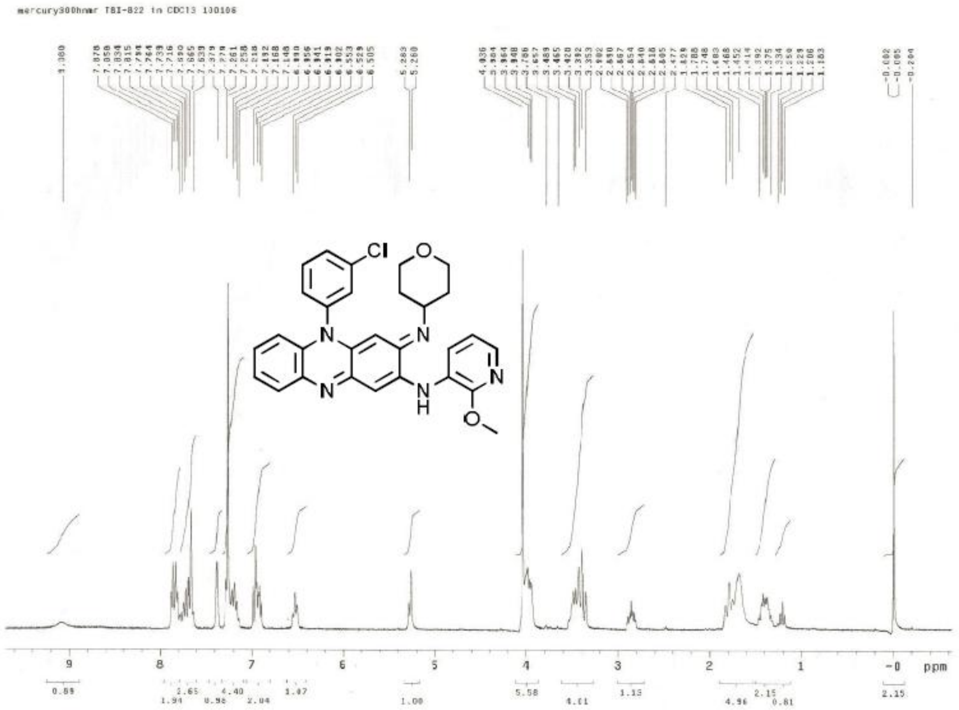

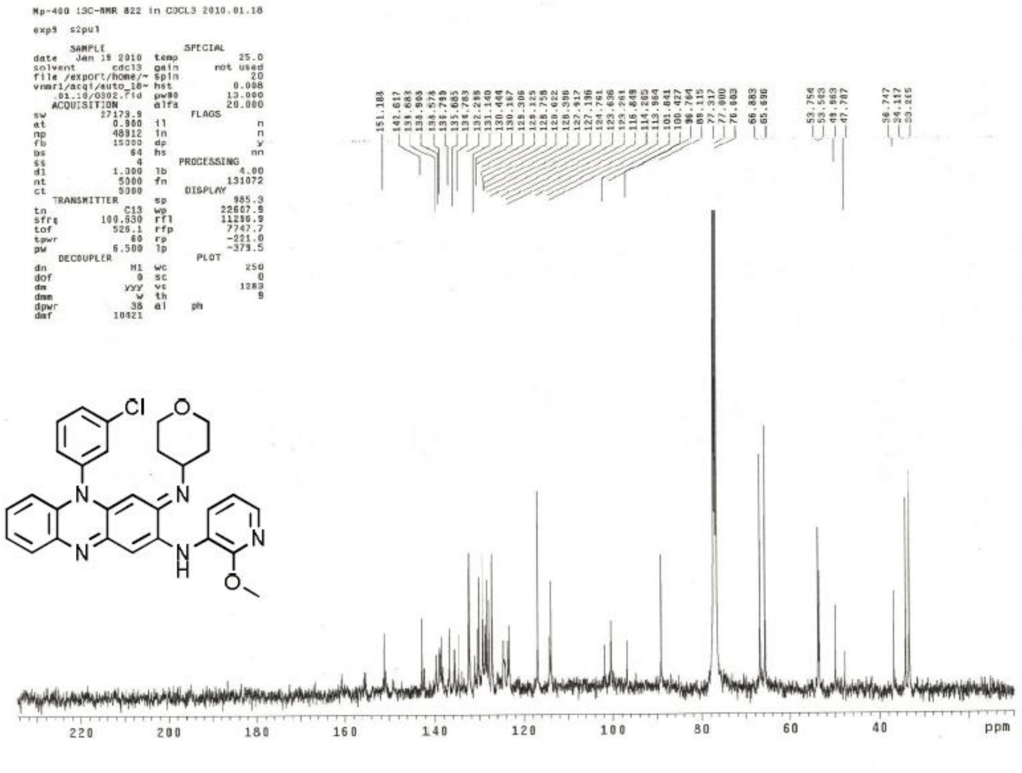

5-(3,4-Difluorophenyl)-3-isopropylimino-2-(2-methoxy-3-pyridyl)amino-3,5-dihydrophenazine (**15**)

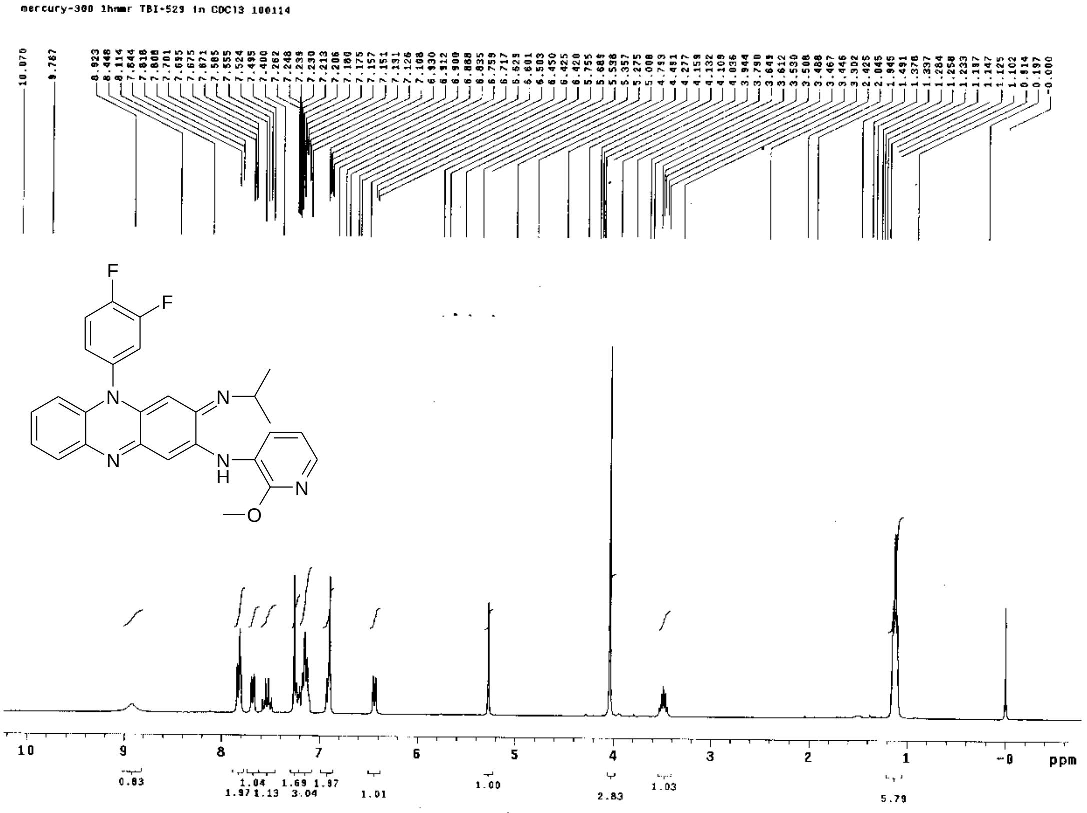

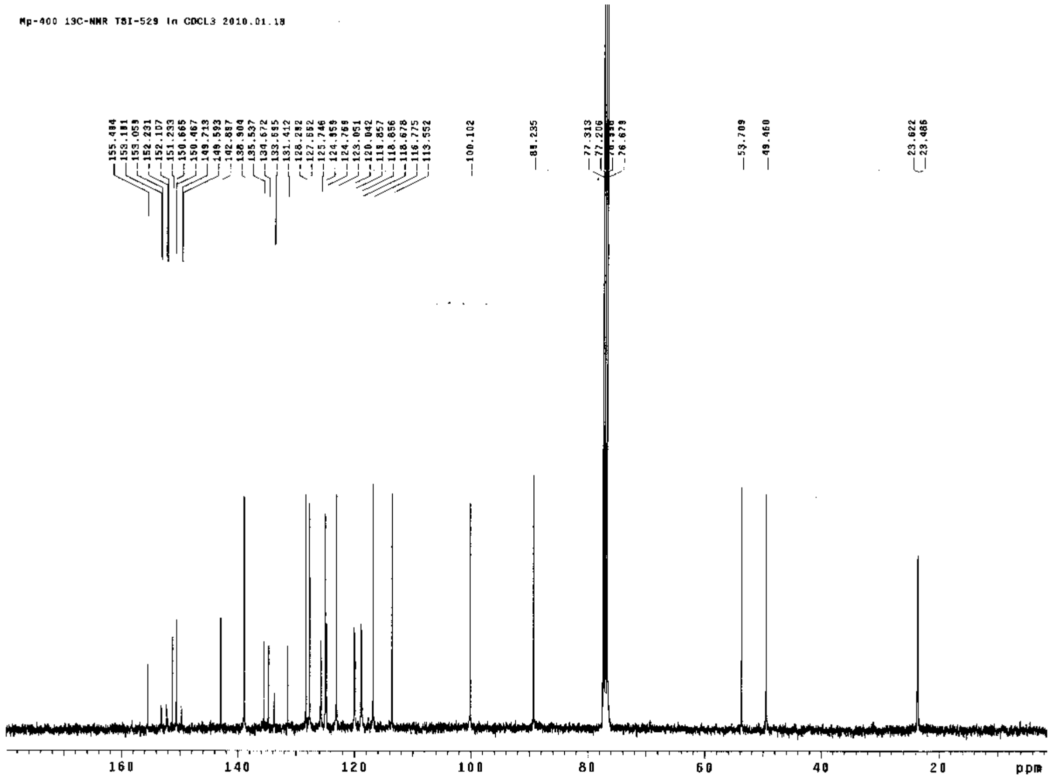

5-(3,4-Difluorophenyl)-3-cyclopropylimino-2-(2-methoxy-3-pyridyl)amino-3,5-dihydrophenazine (**16**)

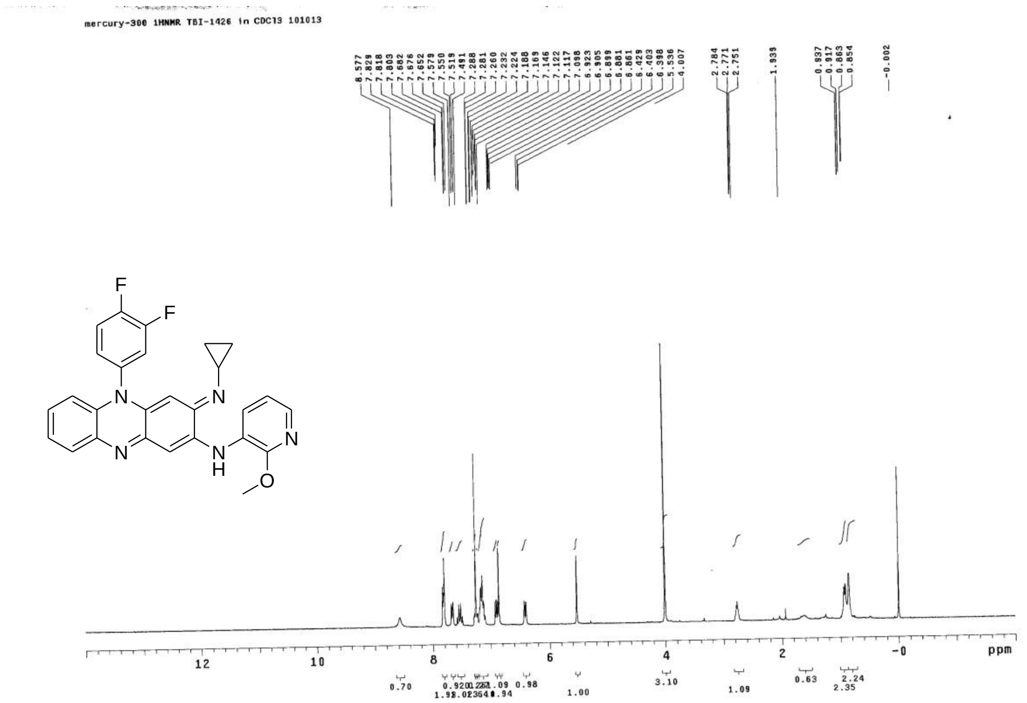

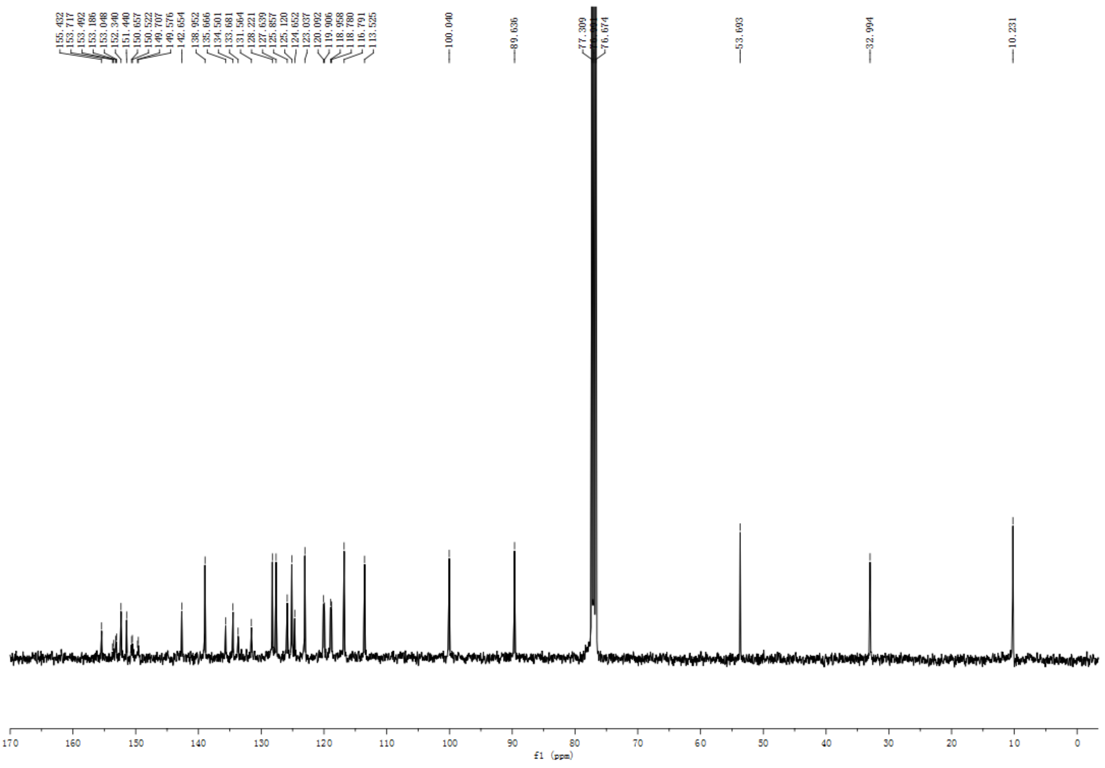

5-(3,4-Difluorophenyl)-3-cyclobutylimino-2-(2-methoxy-3-pyridyl)amino-3,5-dihydrophenazine (**17**)

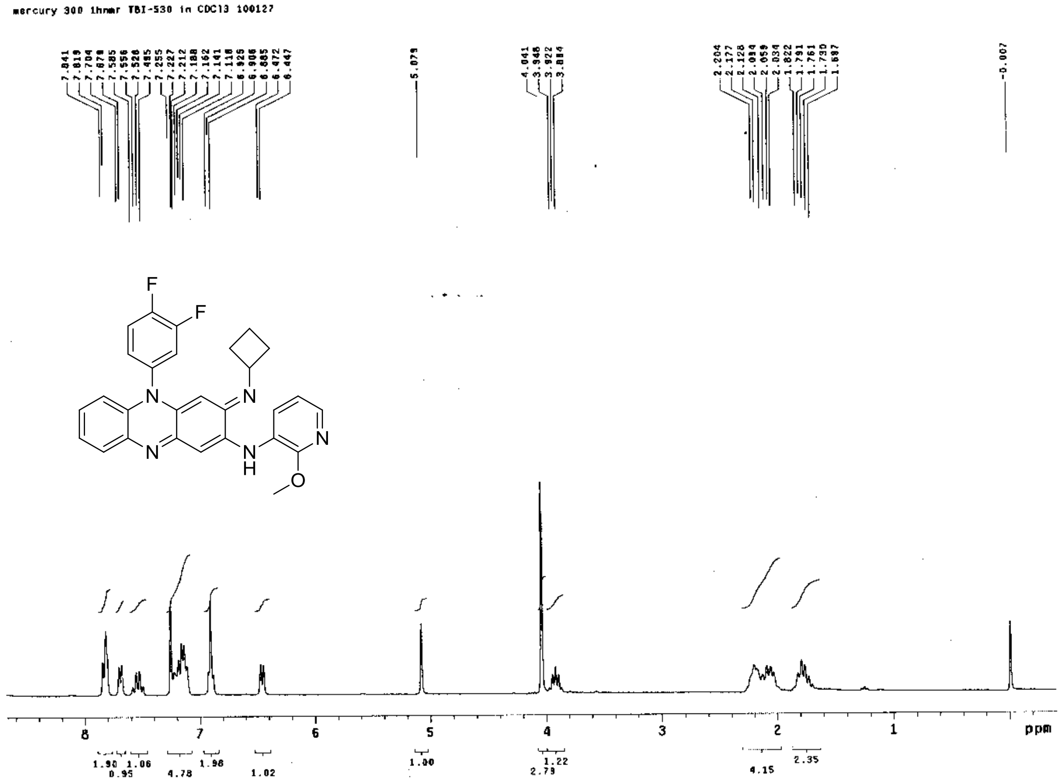

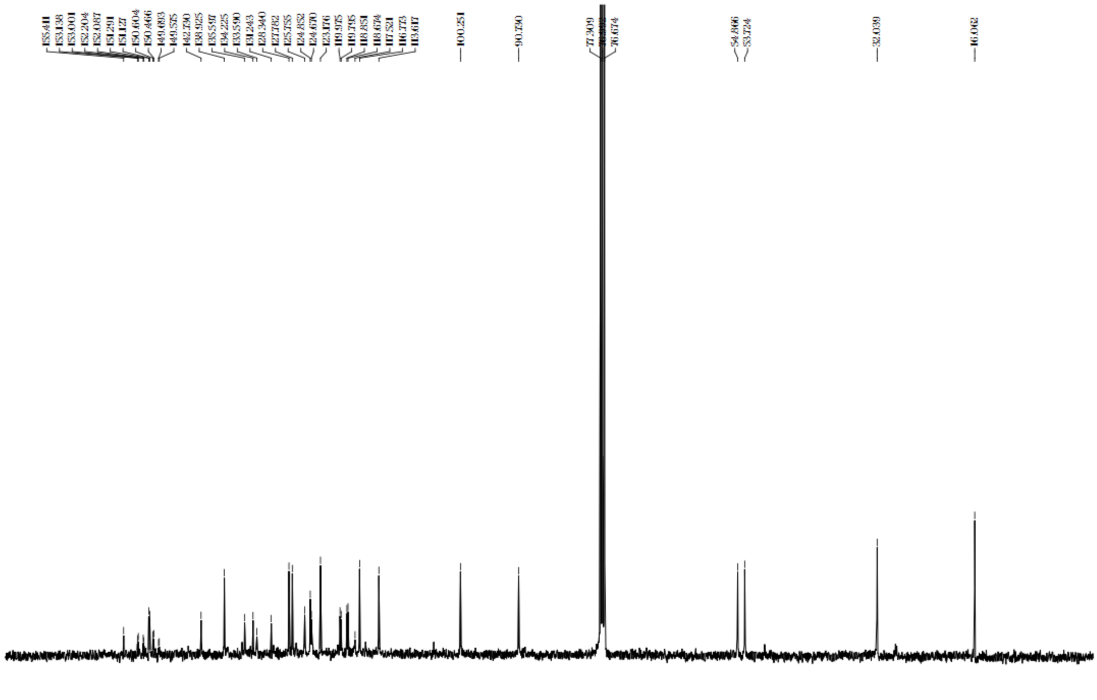

5-(3,4-Difluorophenyl)-3-cyclohexylimino-2-(2-methoxy-3-pyridyl)amino-3,5-dihydrophenazine (**18**)

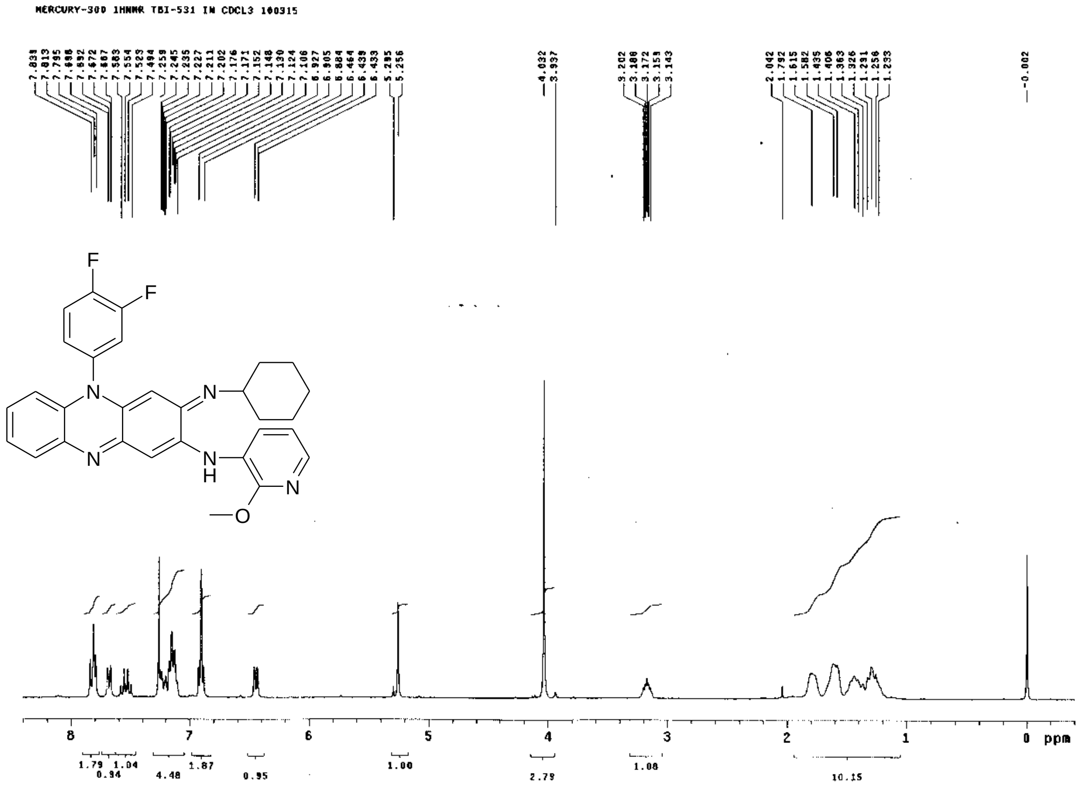

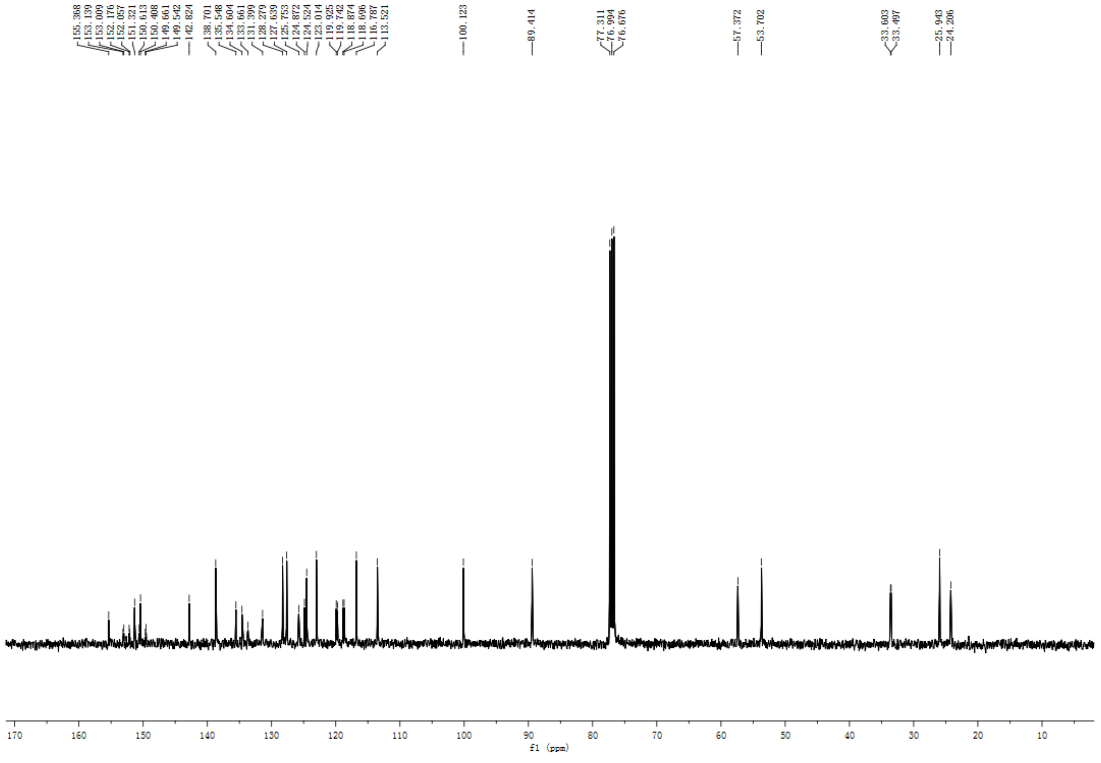

5-(3,4-Difluorophenyl)-3-(4-tetrahydropyranyl)imino-2-(2-methoxy-3-pyridyl)amino-3,5-dihydrophenazine (**19**)

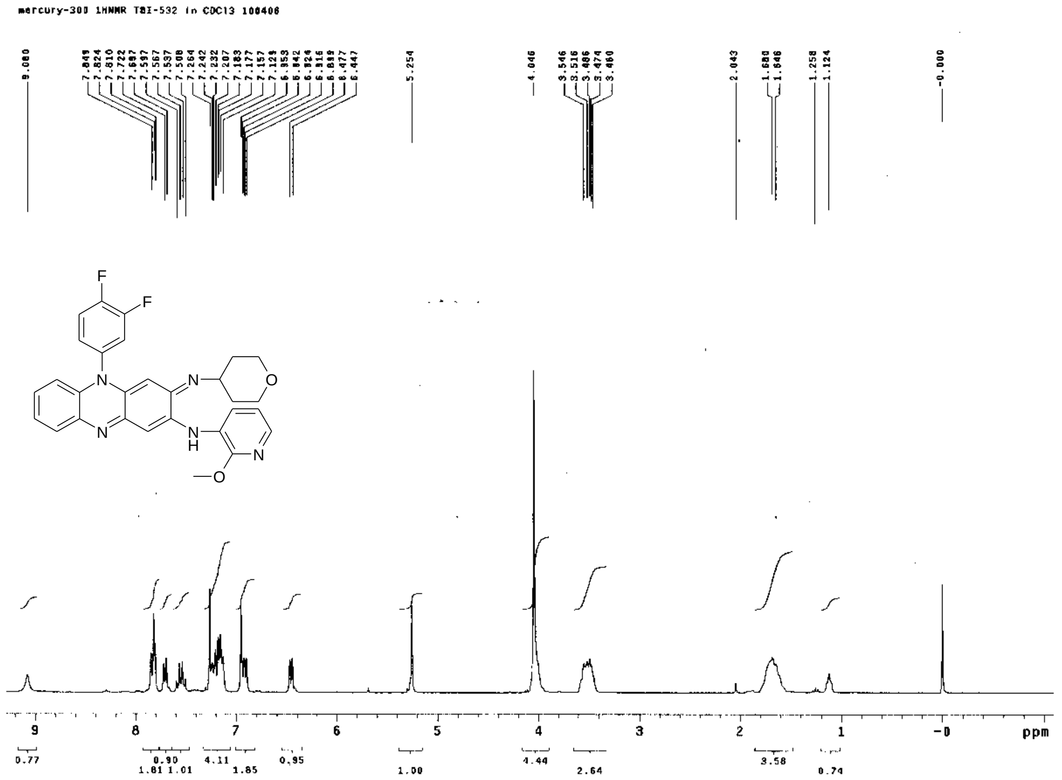

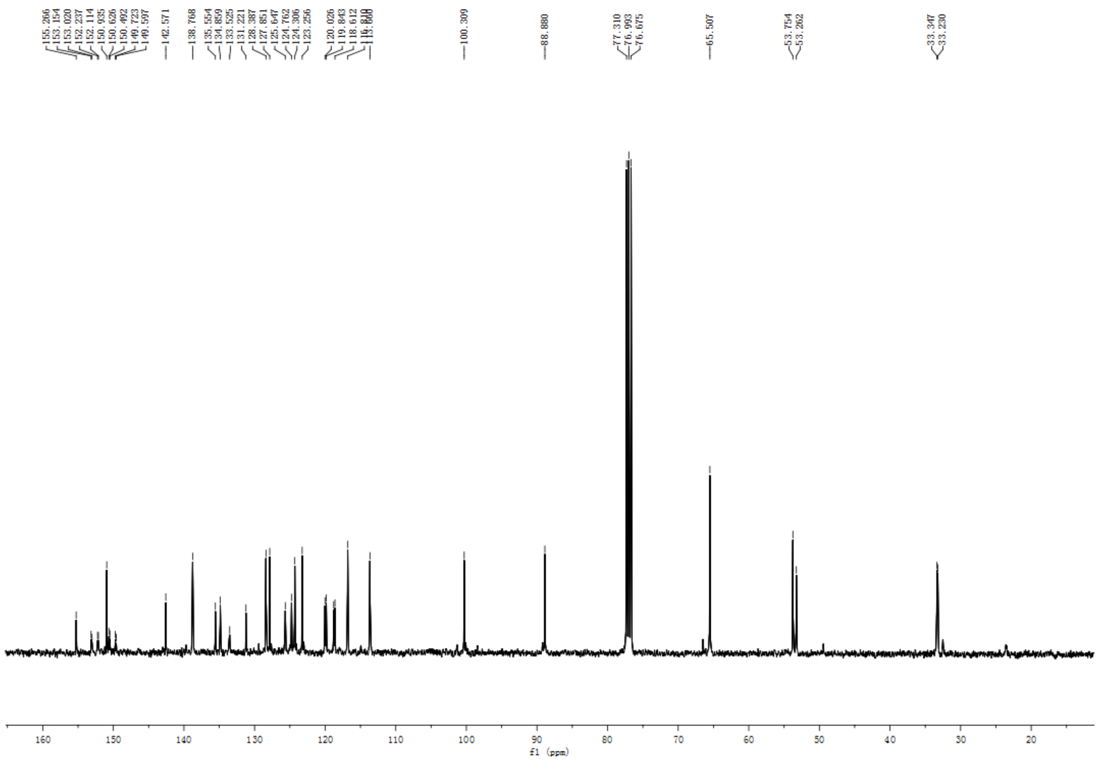

5-(3,4-Difluorophenyl)-3-(4-methoxycyclohexyl)imino-2-(2-methoxy-3-pyridyl)amino-3,5-dihydrophenazine (**20**)

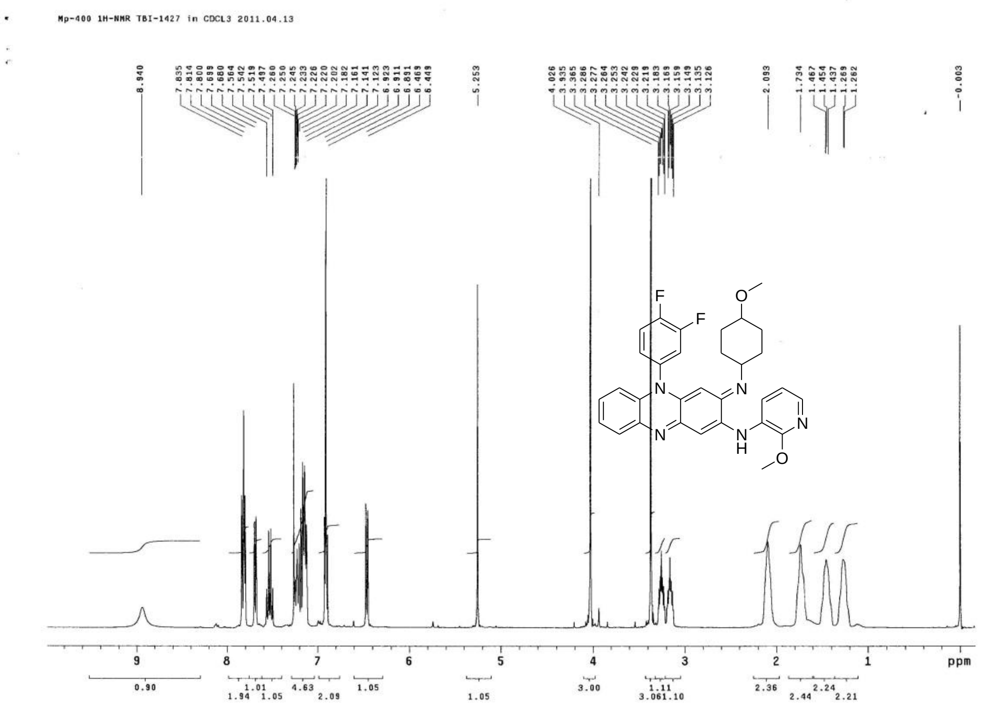

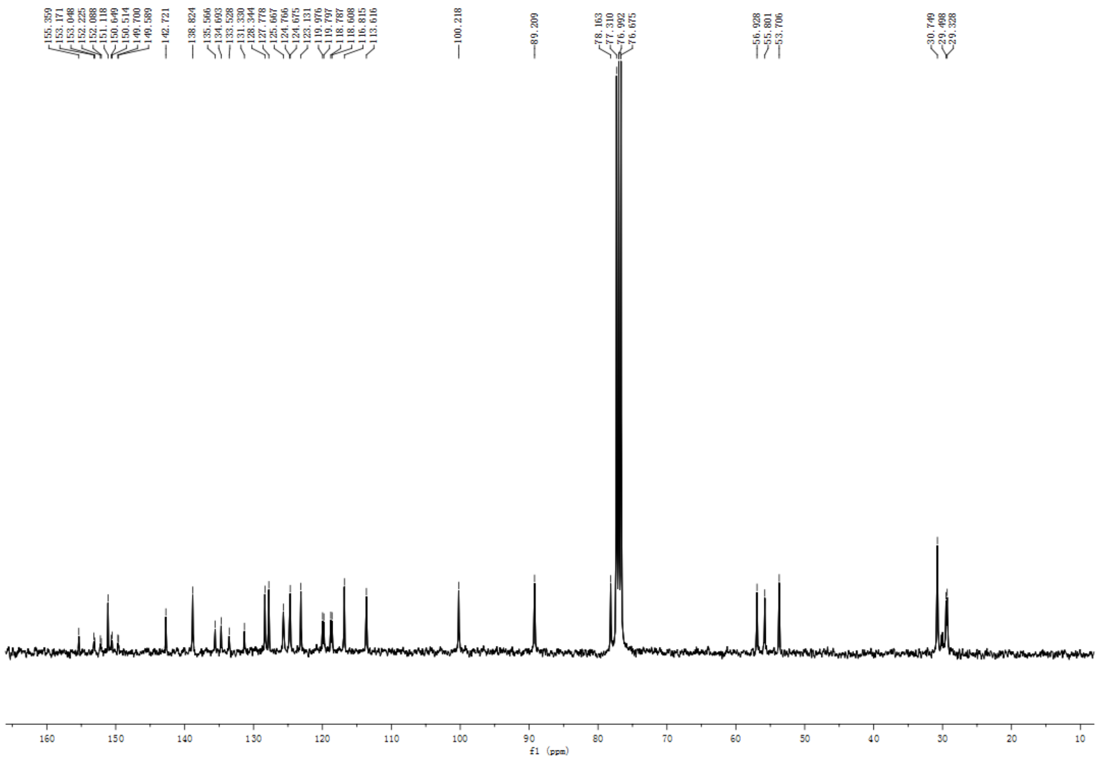

5-(3,4-Dichlorophenyl)-3-isopropylimino-2-(2-methoxy-3-pyridyl)amino-3,5-dihydrophenazine (**21**)

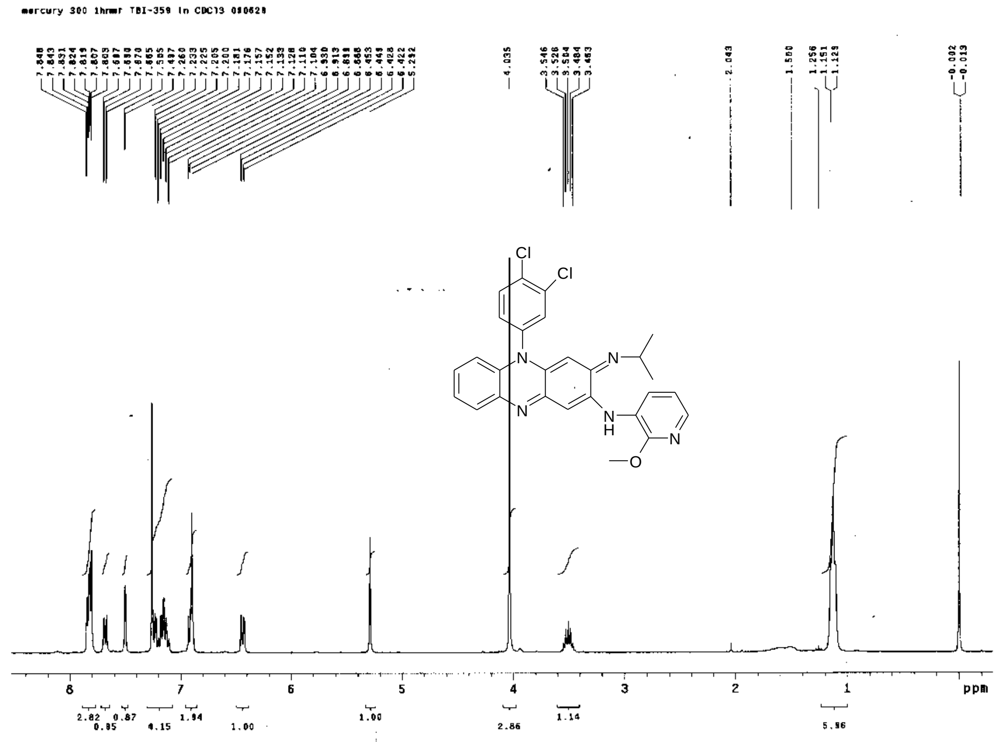

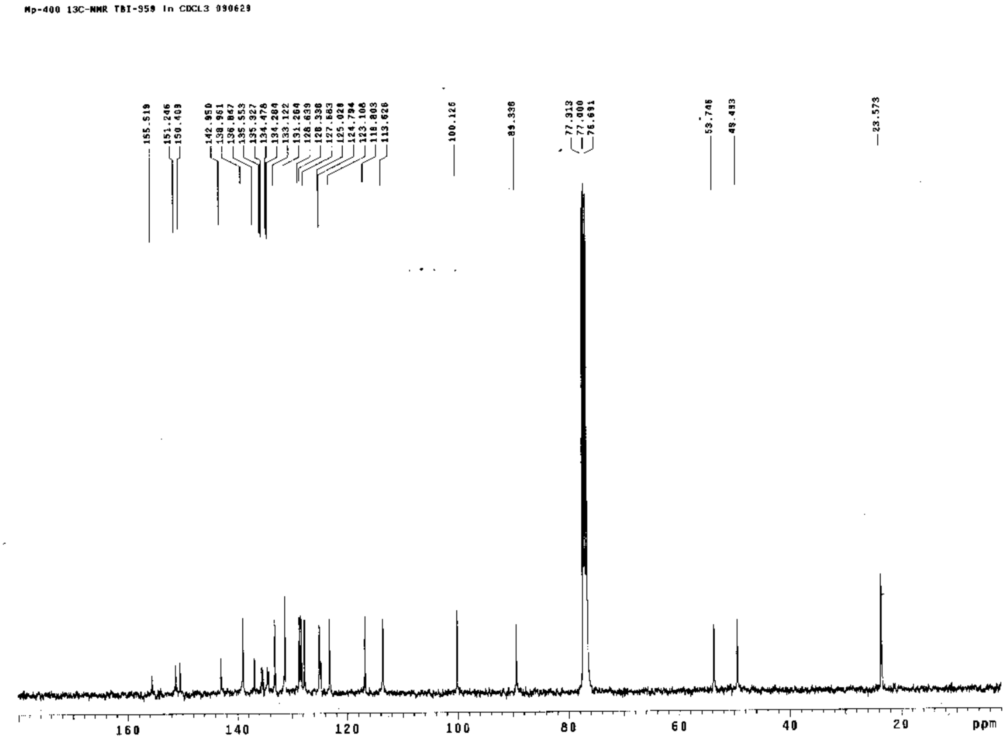

5-(3,4-Dichlorophenyl)-3-cyclopropylimino-2-(2-methoxy-3-pyridyl)amino-3,5-dihydrophenazine (**22**)

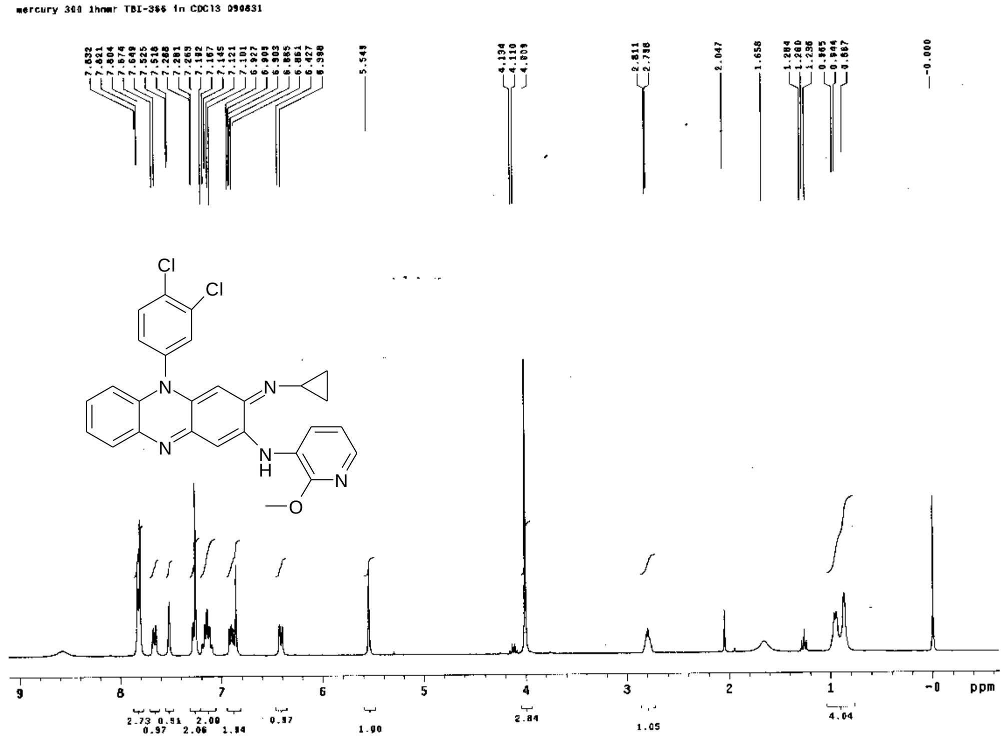

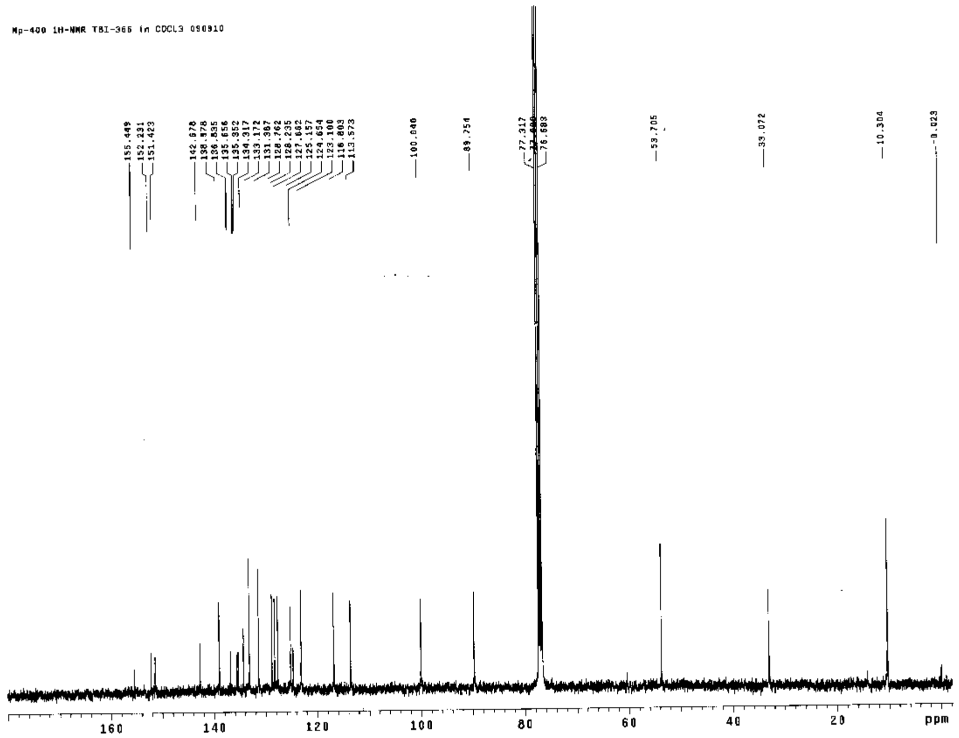

5-(3,4-Dichlorophenyl)-3-cyclobutylimino-2-(2-methoxy-3-pyridyl)amino-3,5-dihydrophenazine (**23**)

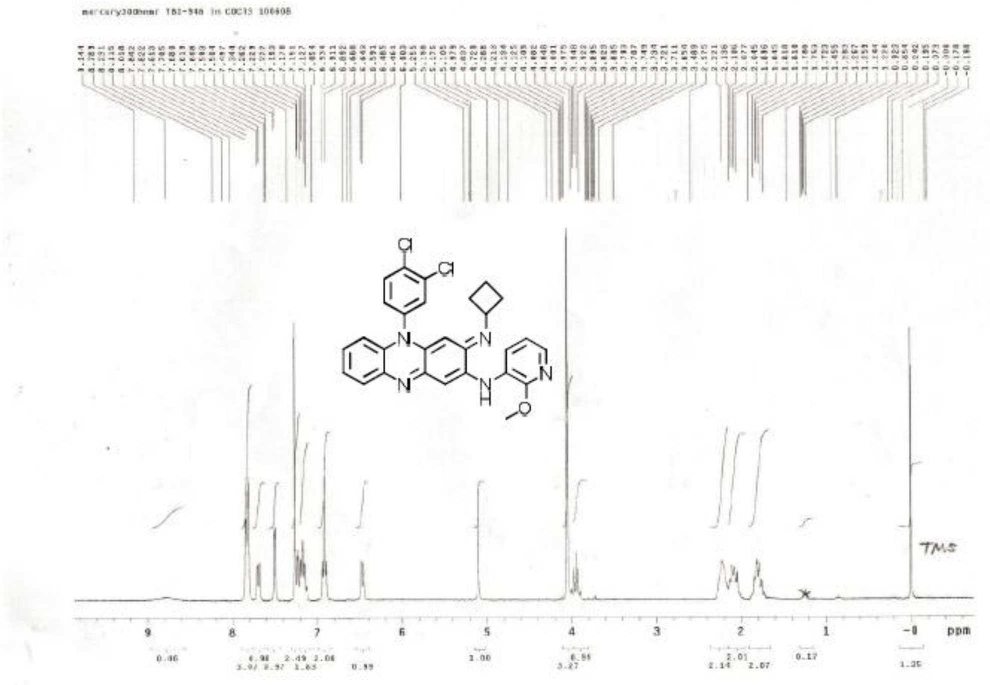

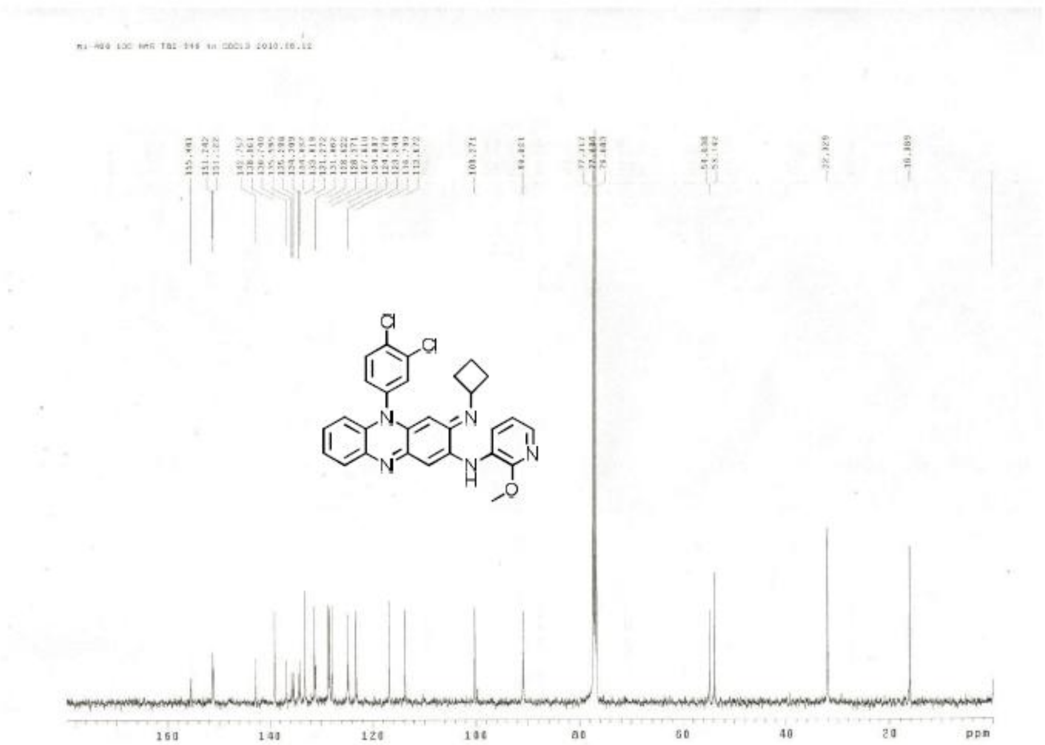

5-(3,4-Dichlorophenyl)-3-(4-tetrahydropyranyl)imino-2-(2-methoxy-3-pyridyl)amino-3,5-dihydrophenazine (**24**)

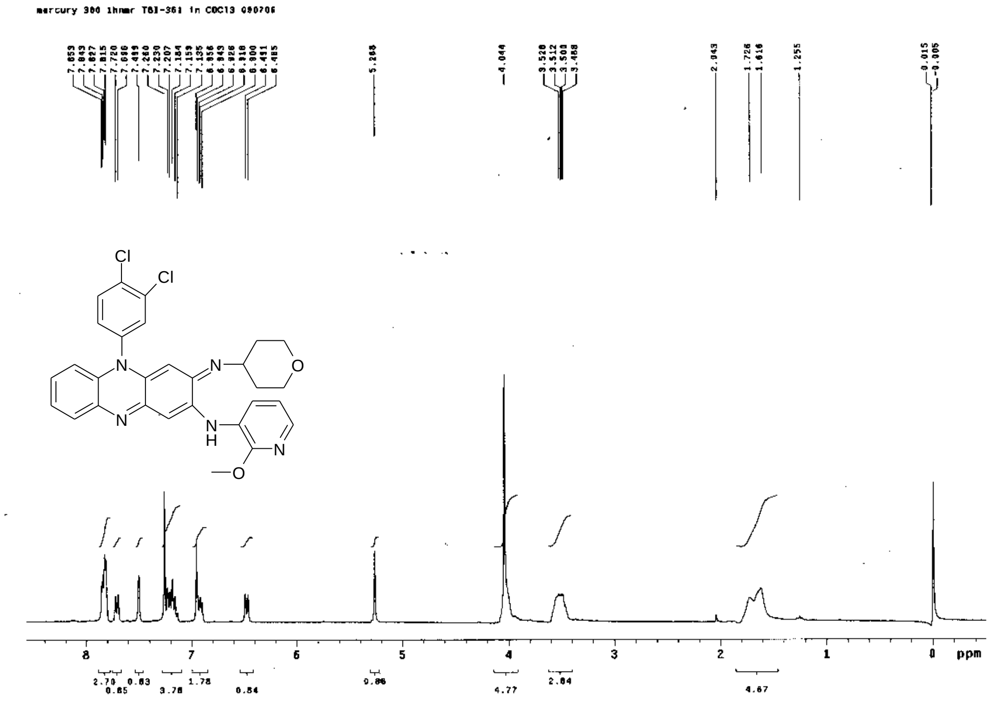

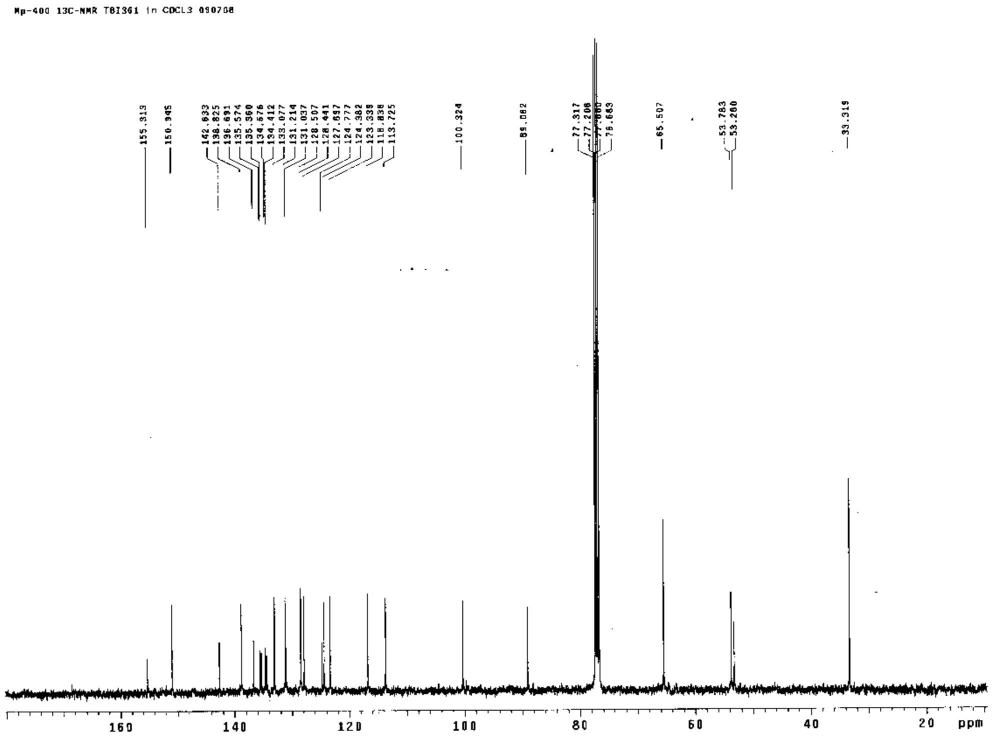

5-(3,4-Dichlorophenyl)-3-(4-methoxycyclohexyl)imino-2-(2-methoxy-3-pyridyl)amino-3,5-dihydrophenazine (**25**)

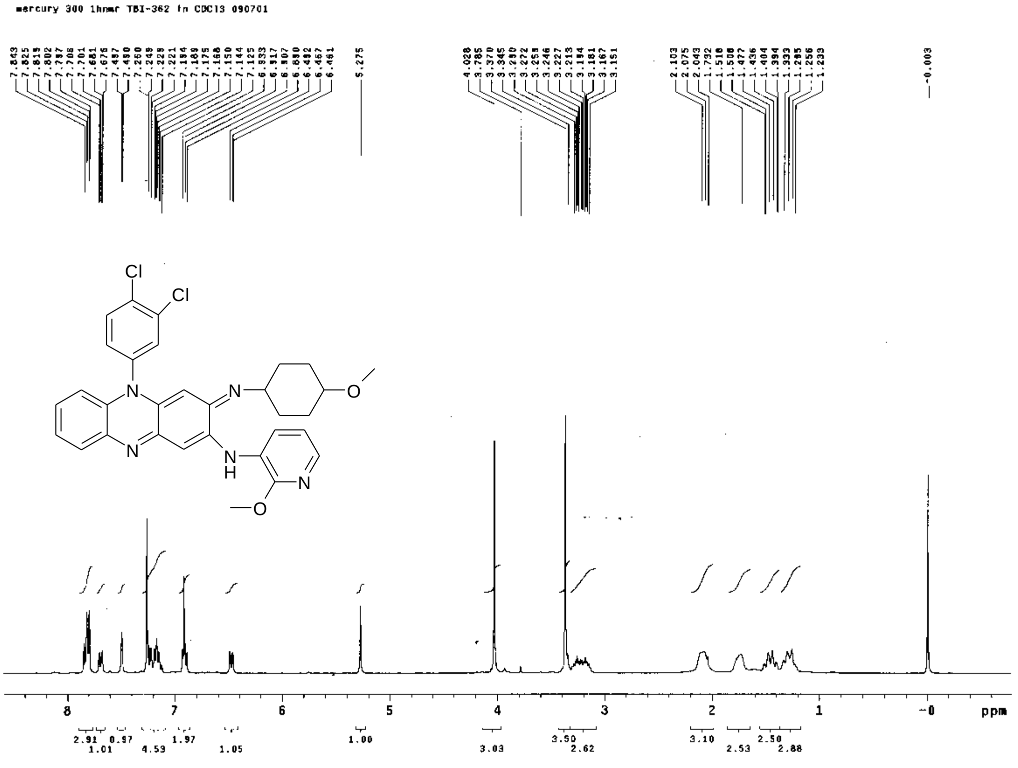

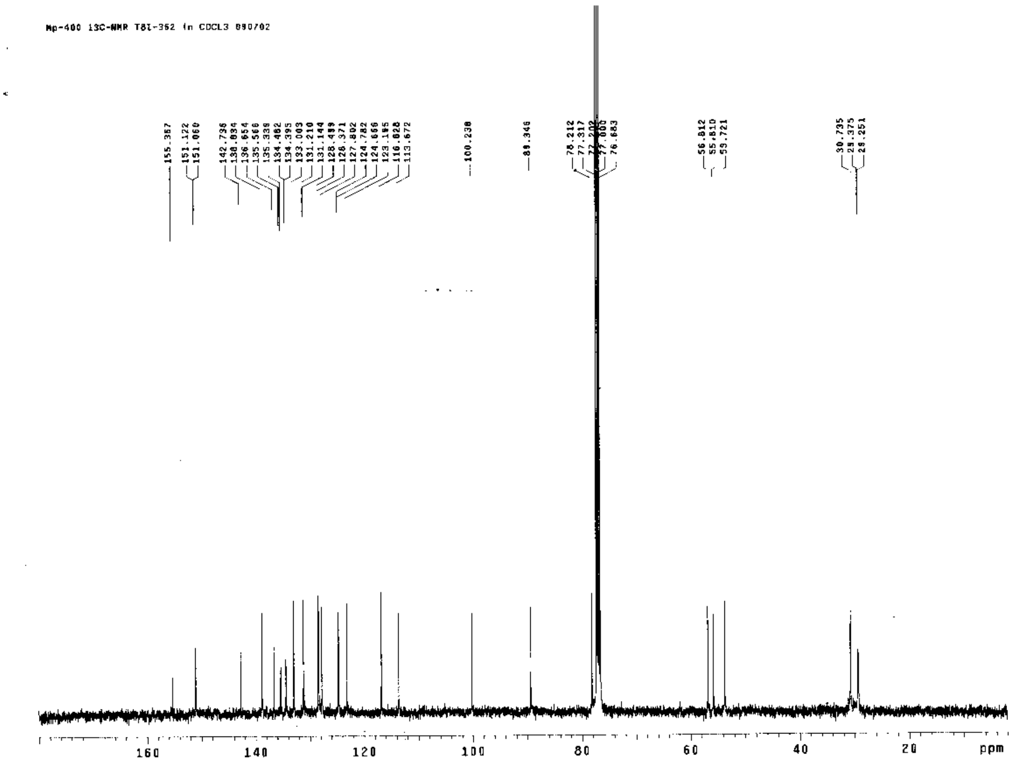

Supplement: Supplementary file 1 [file molecules-19-04380-s001.pdf]
